# Elucidating the mechanism of action of domatinostat (4SC-202) in cutaneous T cell lymphoma cells

**DOI:** 10.1186/s13045-019-0719-4

**Published:** 2019-03-18

**Authors:** Marion Wobser, Alexandra Weber, Amelie Glunz, Saskia Tauch, Kristina Seitz, Tobias Butelmann, Sonja Hesbacher, Matthias Goebeler, René Bartz, Hella Kohlhof, David Schrama, Roland Houben

**Affiliations:** 1 Department of Dermatology, Venereology and Allergology, University Hospital Wuerzburg, Josef-Schneider-Str. 2, 97080 Wuerzburg, Germany; 2 4SC company, Planegg-Martinsried, Germany

**Keywords:** Cutaneous lymphoma, Epigenetic regulation, Histone deacetylase, HDAC, Lysine-specific methylase, LSD1, Tubulin

## Abstract

**Background:**

Targeting epigenetic modifiers is effective in cutaneous T cell lymphoma (CTCL). However, there is a need for further improvement of this therapeutic approach. Here, we compared the mode of action of romidepsin (FK228), an established class I histone deacetylase inhibitor, and domatinostat (4SC-202), a novel inhibitor of class I HDACs, which has been reported to also target the lysine-specific histone demethylase 1A (LSD1).

**Methods:**

We performed MTS assays and flow cytometric analyses of propidium iodide or annexin V-stained cells to assess drug impact on cellular proliferation, cell cycle distribution, and survival. Histone acetylation and methylation as well as caspase activation was analyzed by immunoblot. Gene expression analysis was performed using NanosString technology. Knockdown and knockout of *LSD1* was achieved with shRNA and CRISPR/Cas9, respectively, while the CRISPR/Cas9 synergistic activation mediator system was used to induce expression of endogenous HDACs and LSD1. Furthermore, time-lapse fluorescence microscopy and an in vitro tubulin polymerization assay were applied.

**Results:**

While FK228 as well as 4SC-202 potently induced cell death in six different CTCL cell lines, only in the case of 4SC-202 death was preceded by an accumulation of cells in the G2/M phase of the cell cycle. Surprisingly, apoptosis and accumulation of cells with double DNA content occurred already at 4SC-202 concentrations hardly affecting histone acetylation and methylation, and provoking significantly less changes in gene expression compared to biologically equivalent doses of FK228. Indeed, we provide evidence that the 4SC-202-induced G2/M arrest in CTCL cells is independent of de novo transcription. Furthermore, neither enforced expression of HDAC1 nor knockdown or knockout of LSD1 affected the 4SC-202-induced effects. Since time-lapse microscopy revealed that 4SC-202 could affect mitotic spindle formation, we performed an in vitro tubulin polymerization assay revealing that 4SC-202 can directly inhibit microtubule formation.

**Conclusions:**

We demonstrate that 4SC-202, a drug currently tested in clinical trials, effectively inhibits growth of CTCL cells. The anti-cancer cell activity of 4SC-202 is however not limited to LSD1-inhibition, modulation of histone modifications, and consecutive alteration of gene expression. Indeed, the compound is also a potent microtubule-destabilizing agent.

**Electronic supplementary material:**

The online version of this article (10.1186/s13045-019-0719-4) contains supplementary material, which is available to authorized users.

## Background

Despite recent advances in basic and translational research in the field of cutaneous T cell lymphoma (CTCL) [1, 2], effective treatment options for advanced mycosis fungoides (MF) and Sézary syndrome (SS)—the two most common types of CTLC—are still limited. Usually, conventional therapies result in only short-lived remissions [3–5]. Recent genomic analysis revealed aberrations affecting apoptosis [6], cytokine, and T cell receptor signaling [7] as well as epigenetic regulation [8] as possible drivers in CTCL. In particular, mutations in genes coding for proteins involved in histone modification (acetylation, methylation and ubiquitination) as well as chromatin remodeling were commonly detected [8]. Moreover, hyper-methylated promotor regions of tumor suppressor genes like p16^INK4A^ [9, 10] and hypo-acetylated histones [11, 12]—both leading to silencing of respective promoters—as well as frequently observed overexpression of epigenetic modifiers such as histone deacetylases (HDACs) [13] suggested that targeting of epigenetic events in general and especially inhibition of HDACs may be a feasible therapeutic approach in CTCL [14]. Indeed, two class I HDAC inhibitors, i.e., vorinostat and romidepsin (FK228), achieved good clinical efficacy with objective responses of 25–30% leading to the FDA approval of both drugs for the treatment of CTCL in 2006 and 2009, respectively [15, 16].

While histone acetylation is generally associated with promoter activation, the role of another epigenetic modification, i.e., histone methylation, in regulation of gene expression is more complex and highly dependent on the specific histone residues modified [17]. In this regard, tri-methylation of lysine 9 or lysine 27 of histone H3 (H3K9me3 or H3K27me3) facilitates gene repression while di- or tri-methylation of lysine 4 of histone H3 (H3K4me2 or H3K4me3) mediates promoter activation, whereas mono-methylation of H3K4 is a hallmark of active distal enhancer regions [18].

The histone methylation status is controlled by a balanced action of various methyltransferases and demethylases [19]. Among these enzymes the first histone demethylase to be discovered was the lysine-specific histone demethylase 1a (LSD1), also known as lysine(K)-specific demethylase 1A (KDM1A). LSD1 demethylates mono- and di-methylated H3K4, thereby repressing transcription [20]. This gene-repressing function exerts LSD1 in particular as part of the multi-protein CoREST complex, which besides several other proteins, includes also HDAC1 and HDAC2 and mediates promoter silencing in a multi-step process involving consecutive histone-deacetylation and H3K4-demethylation [21]. In a different context, however, LSD1 may also contribute to transcriptional activation by demethylating the gene-repressive tri-methylated H3K9 [22]. This requires different binding partners [22–24] and might be restricted to a certain splice variant carrying an additional exon [25]. Furthermore, non-histone targets of LSD1 have been described and in the context of tumor biology, it is of special interest that LSD1 can demethylate di-methylated lysine 370 on p53 thereby inhibiting transcriptional activity of this tumor suppressor protein [26].

In various cancers increased expression of LSD1 has been documented and correlates with poor differentiation, higher aggressiveness, epithelial-to-mesenchymal transition, and adverse clinical outcome [27–32]. Therefore, inhibition of LSD1 is considered as a promising antitumor strategy [33–35], and its well-defined active site cavity allowed the design of small molecule inhibitors, which are now evaluated in pre-clinical and clinical studies [36–38]. Notably, due to the functional interplay of LSD1 and HDAC1/2 in the CoREST complex, it has been suggested that combined targeting of HDACs and LSD1 might be superior with respect to cancer-specific cytotoxicity compared to individual inhibition of histone acetylation or methylation [39]. Indeed, synergistic effects of combined LSD1/HDAC inhibition could be demonstrated, and several single drugs inhibiting HDACs as well as LSD1 have been developed [39–42]. One such compound which has been reported to target class I HDACs as well as LSD1 is domatinostat (4SC-202) [43], which is currently investigated in a phase I clinical trial in hematological neoplasms with up to now excellent tolerability and good efficacy [44].

The goal of this study was to analyze in vitro how the combined HDAC/LSD1-inhibitor 4SC-202 affects CTCL cells in comparison to the approved therapeutic HDAC-inhibitor FK228. We find that both substances effectively induce cell death in a set of CTCL cell lines with the difference that cell death only in the case of 4SC-202 is preceded by a G2/M arrest. We provide evidence that this difference is not a consequence of targeting LSD1 by 4SC-202. The 4SC-202 anti-cancer activity is, apart from targeting histone-modifying enzymes, rather based on directly affecting tubulin polymerization.

## Methods

### Cell culture

To study in vitro the impact of FK228 (Selleckchem) and 4SC-202 (4SC AG) on cutaneous lymphoma cells, six different CTCL cell lines were used: CRL-2105 (Synonym: HH, Accesssion: CVCL_1414) [45], CRL-8294 (MJ, CVCL_1414) [46], HTB-176 (H9, CVCL_1240) [47], HuT 78 (NCI-H78, CVCL_0337) [48], MyLa [49], and Se-Ax (SeAx, CVCL_5363) [50]. In addition, HeLa (cervix carcinoma cell line), WaGa (Merkel cell carcinoma), UACC-257 (melanoma), U2OS (osteosarcoma), MCF7 (breast adenocarcinoma), A549 (lung adenocarcinoma), Maver-1 (mantle cell lymphoma), and the human embryonic kidney-derived HEK293T cell line were used in this study. All cell lines were maintained in RPMI supplemented with 10% fetal calf serum (FCS), 100 U/ml penicillin, and 0.1 mg/ml streptomycin. Peripheral blood lymphocytes (PBLs), a dermal fibroblast preparation derived from the skin of an adult donor (fibroblasts A) as well as human foreskin fibroblasts (fibroblasts B), served as untransformed control cells. PBLs were freshly isolated from the blood of a healthy donor by Ficoll-Hypaque density-gradient centrifugation. In vitro doubling times of the different primary cells and cell lines were estimated by cell counting and are summarized in Additional file 1: Table S1.

### MTS assay

In order to measure cell viability and cellular metabolic activity, MTS assay was performed according to standard protocols. Cells were seeded as triplicates in a 96-well plate with 5000 or 15,000 cells/well. FK228 (Selleckchem) or 4SC-202 (4SC AG) were added in incremental concentrations. Culture medium served as negative control. After 72 h, 10 μl MTS reagent was added per well and absorbance was measured at 492 nm (with reference wavelength of 630 nm) after 60 min. IC50 doses were calculated by nonlinear fitting of MTS curves using GraphPad Prism Version 7.0.

### DNA staining

For DNA staining, cells were fixed using ice-cold ethanol (90%) for at least 1 h followed by treatment with a propidium iodide solution (PI) (phosphate buffered saline supplemented with 1% FCS, 0.1 mg/ml PI, and 0.1 mg/ml RNAse A) at 37 °C for 1 h. Cellular DNA content was then analyzed by flow cytometry.

### Annexin V assay

An Annexin V Phycoerythrin conjugate (Annexin V Apoptosis Detection Kit, BD Biosciences) was applied to identify apoptotic cells by flow cytometry according to the manufacturer’s instructions. By double staining with 7-AAD, early apoptotic cells can thereby be identified as 7-AAD^−^/Annexin V^+^ cells while the 7-AAD^+^/Annexin V^+^ double positive population marks the late apoptotic cells.

### Immunoblot

Immunoblotting was performed as previously described [51]. Mouse-derived primary antibodies, anti-HDAC1 (10E2, Cell Signaling, 1:1000), anti-H3K9ac (1B10, Active Motif, 1:1000), anti-β-tubulin (TUB 2.1, Santa Cruz Biotechnology, 1:1000), anti-β-actin (AC-15, Sigma Aldrich, 1:3000), and anti-Caspase-3 (3G2, Cell Signaling, 1:1000), were used in this study while anti-LSD1 (C69G12, Cell Signaling, 1:1000), anti-H3K4me2 (Y47, Abcam, 1:1000), and anti-Cleaved Caspase-3 (D175, Cell Signaling, 1:1000) were rabbit antibodies.

### Real time PCR

Expression levels of *LSD1* and *HDAC 1*-*3* genes were determined by qPCR with SYBR Green technology. RNA was isolated as described in the instruction manual of the peqGOLD Total RNA Kit® (Peqlab), transcribed into cDNA by SuperScript II, and amplified by the primers given in Additional file 1: Table S2. Expression of the target genes was depicted as ∆Ct (target-RPLP0).

### NanoString nCounter® analysis

Alterations of gene expression under treatment with 4SC-202 or FK228 were assessed by NanoString nCounter® analysis (NanoString technologies). One hundred nanograms total RNA were subjected to hybridization with the NanoString kinase Kit (Kinase_V2_Panel-48rxn Kit, NanoString technologies) containing probes for 519 kinase and six housekeeping genes. Following nCounter digital reading the values were globally normalized according to the manufacturer’s protocol.

### Time-lapse microscopy

Since live cell imaging turned out to be not feasible with suspension cells such as CTCL cell lines, adherent histone H2B-GFP and additionally RFP-tubulin expressing HeLa cells were used as a representative model for time-lapse microscopy. Cells were seeded into 4-well slides (ibidi®) in phenol red-free medium, and placed in a live cell imaging chamber that assured standard culture conditions (37 °C, 95% humidity, 5% CO2). Images were taken every 10 to 20 min using Eclipse Ti (Nikon).

### Lentiviral LSD1 knockdown and knockout

To knockdown LSD1, we first generated a selectable lentiviral one-vector system which allows Golden Gate cloning of an shRNA coding sequence under the control of a Doxycyclin (Dox)-inducible promoter (induc shRNA EYFP-P2A-Puro; Genbank: MH749464). As shRNA target sequence for *LSD1,* we used AGGCCTAGACATTAAACTGAA. Lentiviral supernatants were produced as previously described [52]. MyLa cells were infected and following puromycin selection, shRNA expression was induced by addition of 1 μg/ml Doxycyclin.

To achieve an LSD1 knockout, we first cloned an oligonucleotide sequence targeting CGCGGAGGCTCTTTCTTGCG in exon 1 of the *LSD1* gene into the LentiGuide-BSD vector, which had been derived from LentiGuide-Puro [53] (kind gift from Feng Zhang; Addgene plasmid #52963) by replacing the puromycin with a blasticidin resistance. Virus generated with this LentiGuide-BSD-LSD1 construct was used to infect HeLa cells stably expressing Cas9, due to prior transduction with the lentiviral pcdh puro Cas9 followed by Puromycin selection. Blasticidin-resistant single-cell clones were established, and clones lacking LSD1 expression were identified by immunoblot. Knockout was confirmed by sequencing of the genomic region targeted by the *LSD1* guide RNA.

### siRNA transfection

siRNAs targeting HDAC1 and HDAC3 were purchased from Sigma (Mission® esiRNA) and transfection was performed with Lipofectamine® RNAimax (Thermofisher) according to the manufacturer’s instructions.

### In vitro tubulin polymerization assay

An in vitro tubulin polymerization assay kit (Merck) containing > 99% pure bovine tubulin was applied according to the manufacturer’s instructions. Spontaneous formation of microtubules in the presence of GTP at 37 °C was monitored by measuring the OD at 340 nm in an absorbance microplate reader over time.

### Whole cell analysis of tubulin polymerization

To measure the degree of intracellular tubulin polymerization by flow cytometry we performed tubulin staining under conditions where only polymerized tubulin is retained in the cells while tubulin monomers tend to get lost during the fixation procedure [54]. To this end, cells were fixed in 1 ml of 0.5% glutaraldehyde in microtubule-stabilizing buffer (80 mM Pipes [pH 6.8], 1 mM MgCl_2_, 5 mM EDTA, and 0.5% Triton X-100 [TX-100]). After 10 min, 0.7 ml NaBH_4_ (1 mg/ml in PBS) was added, and the cells were pelleted by centrifugation. Cells were then re-suspended in 20 μl of PBS containing 50 μg/ml RNase A, 0.2% TX-100, 2% bovine serum albumin [BSA], and 0.1% NaN_3_, and incubated overnight at 4 °C. After additional 3 h of incubation with an Alexa Fluor® 488 anti-Tubulin-α Antibody (clone 10D8; BioLegend), 200 μl of 50 μg/ml propidium iodide in PBS were added followed by flow cytometry. Median tubulin fluorescence was determined for the 4 N fraction.

## Results

### 4SC-202 inhibits growth of CTCL cell lines at low micromolar concentrations

**Fig. 1:**
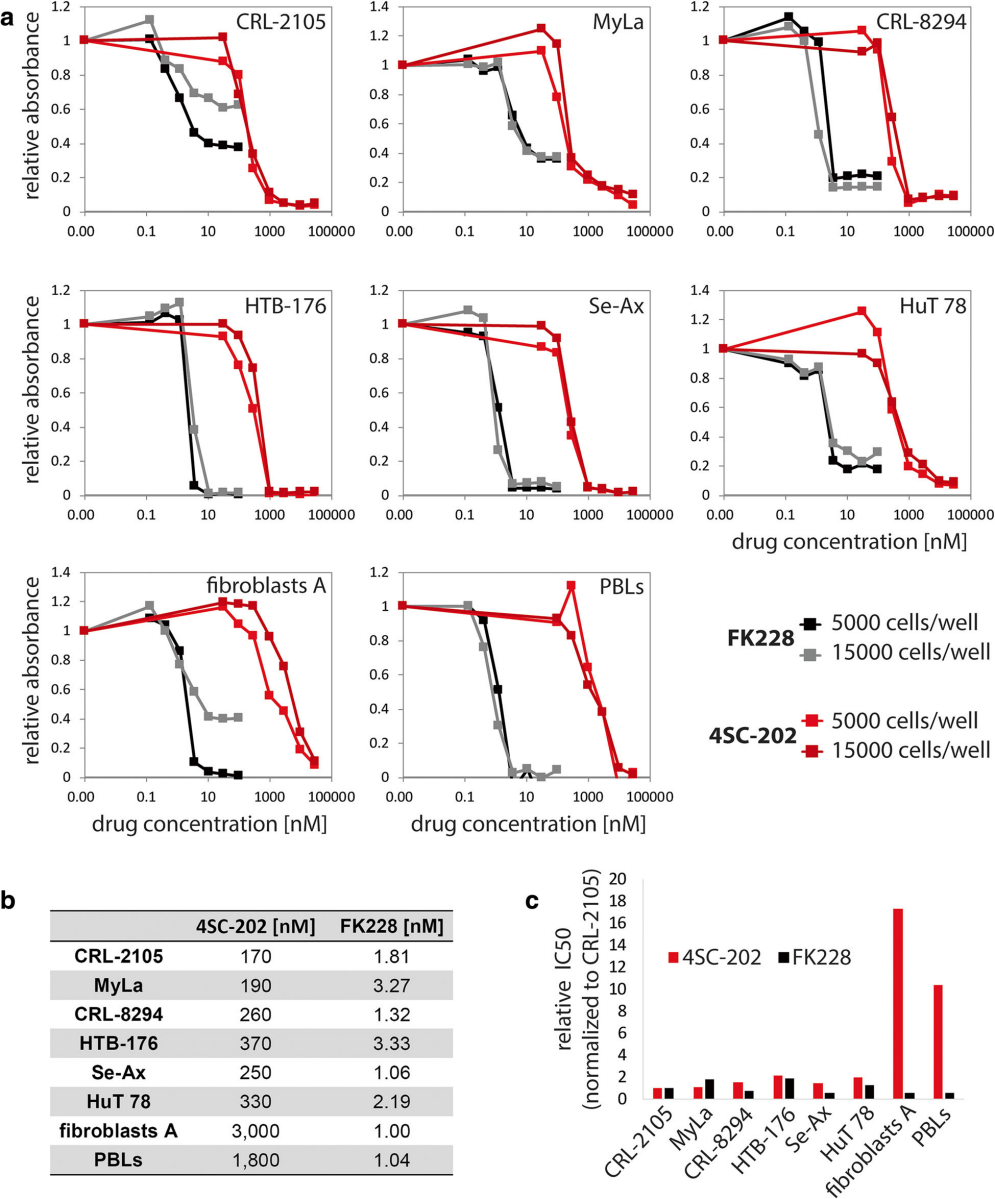
Growth inhibition of CTCL cell lines by 4SC-202 and FK228. **a** Dose response curves for the indicated CTCL cell lines as well as freshly isolated peripheral blood lymphocytes (PBLs) and primary dermal fibroblasts derived from adult skin (fibroblasts A) were determined using the MTS assay. To this end, cells were incubated for 3 days with different concentrations of 4SC-202 and FK228 in triplicates and at two different cell densities. Following incubation with the MTS reagent absorbance at 490 nm was determined and values normalized to untreated controls are depicted. **b**, **c** IC50 doses for 4SC-202 and FK228-treated cells were calculated by nonlinear fitting of the MTS data

FK228 (romidepsin), which received FDA approval for the treatment of CTCL in 2009, has been described to inhibit the class I HDAC members, HDAC1, 2, and 3, at nanomolar levels while being less effective against HDACs 4, 6, 7, 8, and 9 [55, 56]. Similarly, albeit around one log less sensitive, the new orally available benzamide type HDAC inhibitor 4SC-202 is targeting the epigenetically important enzymes HDAC1–3 without affecting other HDAC enzymes at clinically relevant concentrations [57]. Importantly, however, 4SC-202 additionally inhibits a further epigenetic regulator, the lysine-specific demethylase 1 (LSD1) [43, 57], which in concert with HDAC1 and 2 mediates promoter silencing in particular as part of the multi-protein CoREST complex [21]. Because combined targeting of HDACs and LSD1 has been proposed to bear superior anti-cancer capabilities compared to sole HDAC inhibition [39], we started out to compare 4SC-202 with FK228 in six different CTCL cell lines by determining dose response curves. Cells were cultured for 72 h at two different cell densities before viability was analyzed by MTS assay. All CTCL cell lines showed a sharp drop in the MTS signal between 0.1 and 1 μM 4SC-202 while for peripheral blood lymphocytes (PBLs) and fibroblasts used as nonmalignant controls the curves declined over a broader range and were shifted towards higher concentrations (Fig. 1a red curves). Indeed, the calculated IC50 values for 4SC-202 were for almost all CTCL cell lines one order of magnitude lower compared to fibroblasts and PBLs (Fig. 1b and c). Interestingly, this unequal sensitivity was not associated with differential expression of the drug targets, since quantitative mRNA analysis revealed very similar expression levels for *HDAC1*, *3*, and *LSD1* in PBLs, fibroblasts. and CTCL cell lines while for *HDAC2* the expression was generally much lower (Additional file 1: Figure S1). In case of FK228 treatment, we did not observe differences between CTCL cell lines and untransformed fibroblasts and PBLs (Fig. 1). Nevertheless, also FK228 effectively affected growth of the CTCL cell lines, and the absolute concentrations necessary for suppression of CTCL growth were much lower in case of FK228 compared to 4SC-202 (Fig. 1).

### Only 4SC-202 induces an accumulation of cells in the G2/M phase of the cell cycle prior to death

**Fig. 2:**
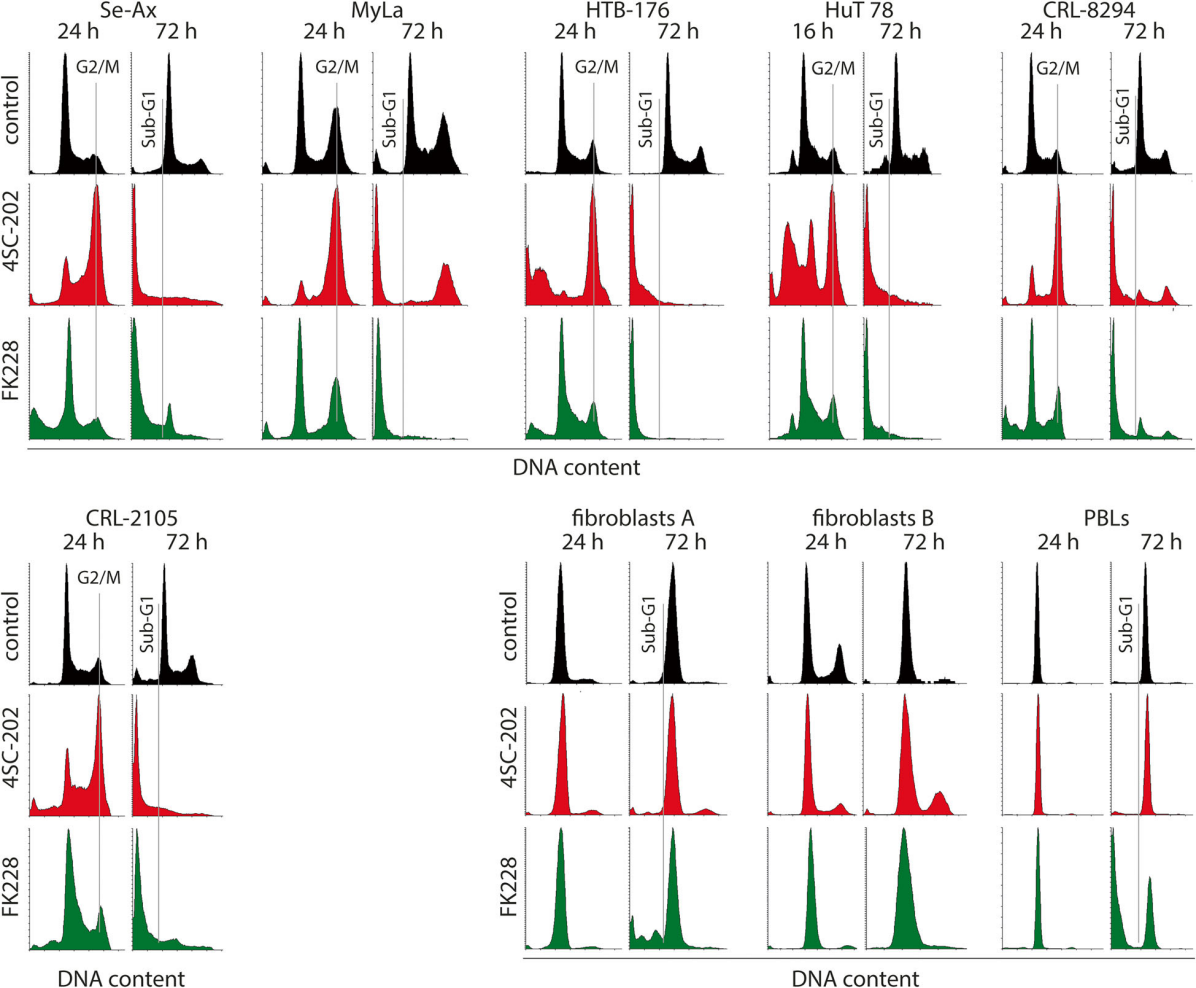
Only cell death induced by 4SC-202 is preceded by a G2/M arrest. Following 1 or 3 days of incubation with 4SC-202 (1 μM) or FK228 (10 nM), genomic DNA of fixed cells was stained with propidium iodide and analyzed by flow cytometry. Besides six CTCL cell lines, three primary untransformed cell cultures were investigated. These nonmalignant controls included freshly isolated peripheral blood lymphocytes (PBLs), primary dermal fibroblasts derived from adult skin (fibroblasts A), and highly proliferative fibroblast derived from juvenile foreskin (fibroblast B)

To further explore how the two drugs affect the cells, we performed DNA staining following 4SC-202 and FK228 treatment. One day of incubation with 1 μM 4SC-202 induced an accumulation in the G2/M phase of the cell cycle in all six CTCL cell lines (Fig. 2). Moreover, 4SC-202-treated HuT 78 and HTB-176 cells presented already an increased sub-G1 fraction at this time point, and this dead cell population became the dominant fraction in all CTCL cell lines after 3 days of 4SC-202 treatment (Fig. 2). Similarly, 10 nM of FK228 induced extensive cell death in the CTCL cells evident by a predominant sub-G1-percentage after 72 h. In contrast to 4SC-202, however, this was not preceded by a G2/M arrest after 24 h of drug exposure (Fig. 2). DNA staining of untransformed control cells revealed weak (fibroblasts) to strong (PBL) cell death induction upon treatment with FK228 for 3 days. When these cells were treated with 1 μM 4SC-202, however, they neither displayed G2/M arrest nor cell death (Fig. 2). The control cells used in the MTS assay (Fig. 1), i.e., PBLs and a fibroblast preparation from adult skin (fibroblasts A), divide much slower than the CTCL cells. Hence, to exclude that, the absence of a 4SC-202-induced mitotic arrest is related to the division rate of the cells, we additionally analyzed foreskin fibroblasts (fibroblasts B), which proliferate comparably fast as most of the CTCL cell lines (Additional file 1: Table S1). Notably, also these fast dividing untransformed cells did not show any accumulation of cells in mitosis when analyzed after 24 h nor was prominent cell death induced upon treatment with 1 or 2 μM 4SC-202 (Fig. 2). In conclusion, 4SC-202 at 1 μM is strongly cytotoxic towards all analyzed CTCL cell lines but not towards untransformed control cells, and induced cell death is—in contrast to FK228—preceded by a G2/M arrest. To test whether 4SC-202 induces G2/M arrest also in other tumor cells, we treated cell lines from six further cancer entities with 2 μM 4SC-202. As depicted in Additional file 1: Figure S2, an accumulation of 4-N cells was evident in all tested cell lines derived from melanoma, Merkel cell carcinoma, breast cancer, lung adenocarcinoma, mantle cell lymphoma or osteosarcoma.

### G2/M arrest and cell death induced by 4SC-202 is associated with only minor changes in acetylation and methylation of histone proteins in CTCL cell lines

**Fig. 3:**
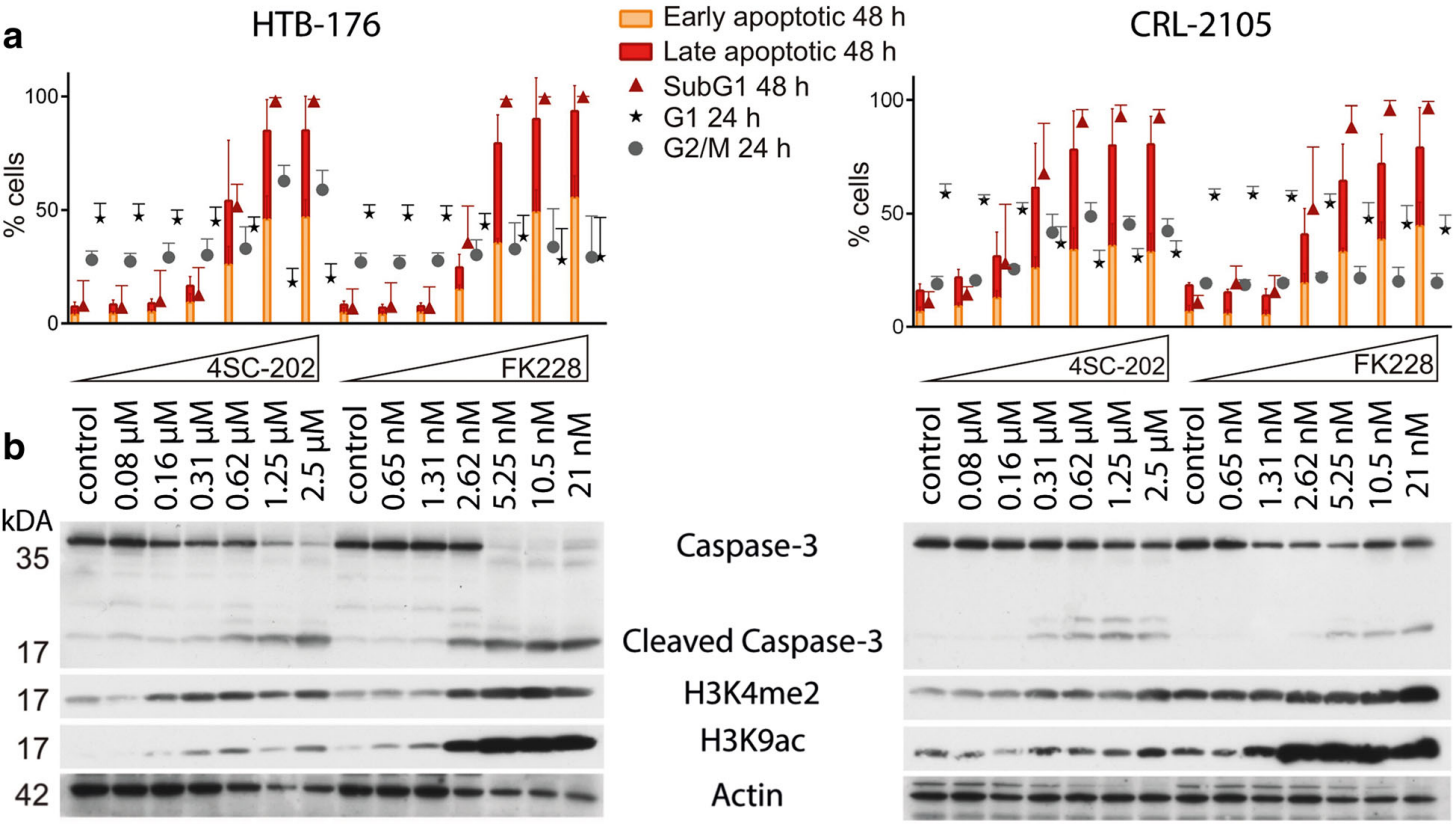
4SC-202 induces G2/M arrest and apoptosis in CTCL cell lines at concentrations hardly affecting acetylation and methylation of histone proteins. CRL-2105 and HTB-176 cells were treated with incremental doses of 4SC-202 or FK228. **a** After 24 and 48 h, cellular DNA content was analyzed by PI staining of fixed cells, and apoptosis was assessed by annexin V/7-AAD double staining. Early and late apoptotic cells were identified as 7-AAD^−^/Annexin V^+^ and 7-AAD^+^/Annexin V^+^, respectively. Mean values (± standard deviation (SD)) of three independent experiments are depicted. **b** Total cell lysates harvested after 48 h of drug treatment were subjected to immunoblot analysis using antibodies recognizing the indicated targets (H3K4me2: histone H3 dimethyl lysine 4; H3K9ac: histone H3 acetyl lysine 9)

We next addressed the question whether the differential response of CTCL cell lines towards the HDAC inhibitor FK228 and the combined HDAC/LSD1 inhibitor 4SC-202 might be due to differences in targeting HDACs and LSD1. To this end, we correlated the pattern of histone acetylation and methylation with the biological response towards the two compounds in two CTCL cell lines. Upon treatment of HTB-176 and CRL-2105 cells with increasing concentrations of 4SC-202 and FK228, we observed an increase in the sub-G1 fraction (Fig. 3a) accompanied by caspase-3 cleavage (Fig. 3b) and the appearance of early apoptotic cells (Annexin V^+^ 7AAD^−^) (Fig. 3a). However, only in the case of FK228, this was associated with a strong increase in acetylation of lysine 9 of histone 3 (H3K9ac) (Fig. 3b). Surprisingly, enhanced di-methylation of lysine 4 of histone 3 (H3K4me2) was also more pronounced in case of FK228 than in response to the designated LSD-1 inhibitor 4SC-202 at concentrations leading to comparable rates of cell death (Fig. 3a and b). As demonstrated before, a G2/M cell cycle arrest occurred only in the 4SC-202-treated cells (Fig. 3a).

Next, we analyzed whether higher concentrations of 4SC-202 could induce more pronounced histone acetylation and methylation in CTCL cells lines. Indeed, H3K9ac and H3K4me2 were strongly enhanced at 3, 10, or 30 μM 4SC-202 reaching levels comparable to FK228 in the three analyzed CTCL cell lines MyLa (Additional file 1: Fig. S3a), HTB-176, and CRL-2105 (Additional file 1: Fig. S4). In contrast, 1 μM 4SC-202, which already induces a full response with respect to G2/M arrest (Fig. 2), led to only very modest increases in both modifications (Additional file 1: Figs. S3a and S4). Interestingly, we observed in MyLa cells that 4SC-202 concentrations associated with strong changes in histone modifications induced even a less pronounced G2/M arrest (Additional file 1: Figs. S3b and c).

### Compared to FK228 only minor changes in gene expression are induced by cytotoxic 4SC-202 concentrations

**Fig. 4:**
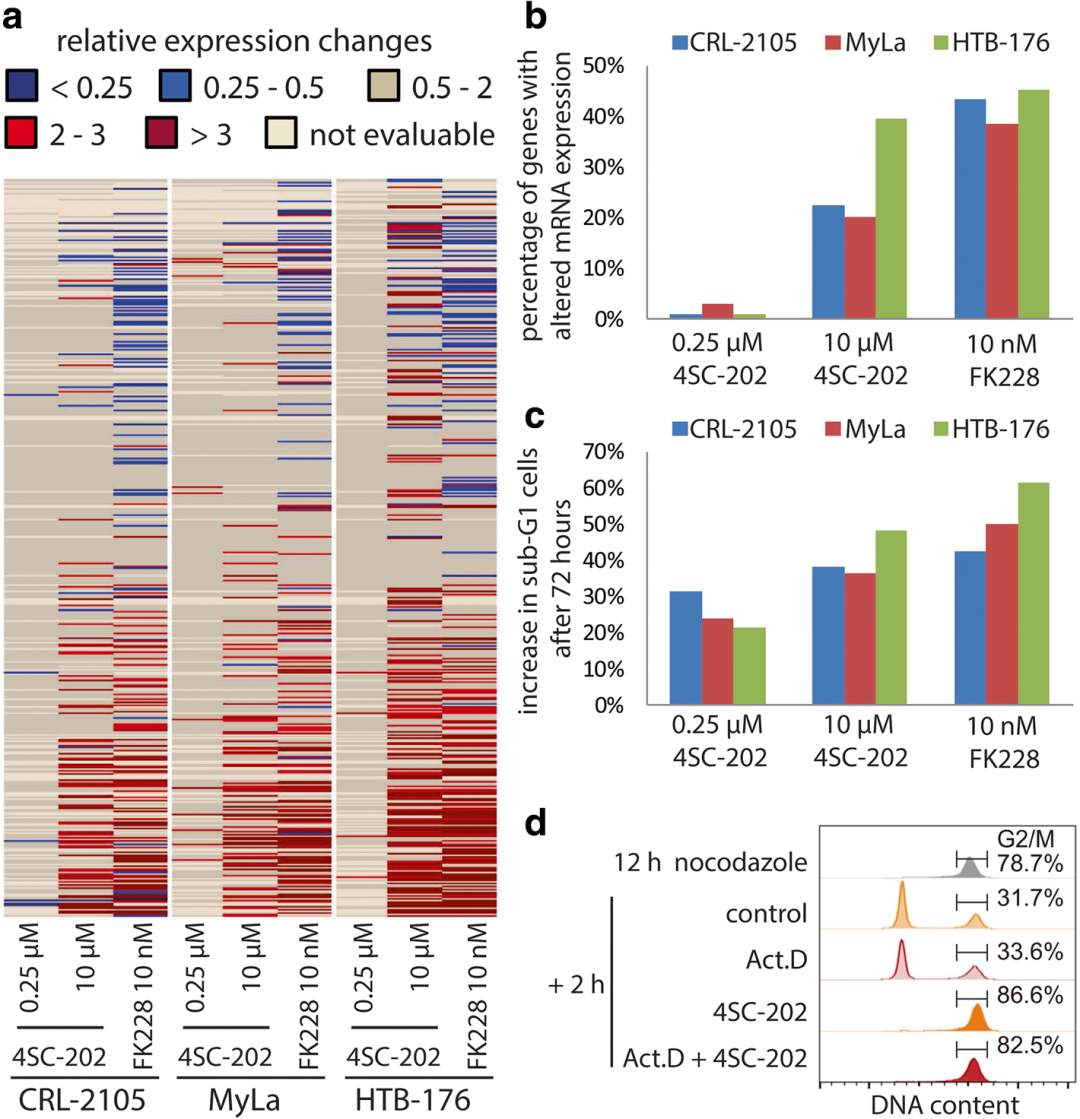
The role of gene expression in 4SC-202-induced arrest and cytotoxicity. **a**, **b** To evaluate the extend of gene expression changes induced by FK228 and different concentrations of 4SC-202, we applied NanoString nCounter® analysis with mRNA derived from the indicated CTCL cell lines after 24-h treatment with 4SC-202 and FK228. A NanoString Panel allowing quantification of mRNA from 519 different kinases and 6 housekeeping genes was used. Following normalization, changes relative to DMSO treated control cells were calculated and are displayed in **a** with the indicated color code. Very low expressed genes (< 0.03% of GAPDH) very excluded from further analysis and are displayed as not evaluable. **b** The percentage of genes that were more than twofold up- or downregulated are depicted. **c** The indicated cell lines were incubated for 72 h with 4SC-202 or FK228. Cellular DNA was stained by propidium iodide and the increase in sub-G1 cells relative to control cells is depicted. **d** To evaluate the necessity of de novo gene expression for the 4SC-202-induced G2/M arrest, HTB-176 cells were first arrested in prometaphase by a 12-h treatment with 100 nM nocodazole (Noc). Then nocodazole was removed and cell cycle progression in the presence or absence of the transcription inhibitor Actinomycin D (Act.D) and/or 4SC-202 was assessed by propidium iodide staining

The proposed modus operandi of how epigenetic modifiers affect cancer cells is through changes in gene expression as a consequence of altered histone modification [14]. Since 4SC-202 could induce profound biological effects in CTCL cells without major changes in relevant histone modifications, we asked how respective drug concentrations would affect gene expression. To this end, we performed NanoString nCounter™ gene expression analyses [58] using an array allowing direct quantification of mRNAs coding for 519 different kinases and eight endogenous controls. In line with the observed differences in histone modification (Fig. 3b), 10 nM FK228 induced over twofold alterations in expression in 39 to 45% of the evaluable genes in the three analyzed cell lines (Fig. 4a and b). In contrast, in response to 0.25 μM 4SC-202—a concentration able to induce G2/M arrest (Additional file 1: Fig. S5) as well as profound cell death (Fig. 4c and Additional file 1: Fig. S5)—only 1–3% of the mRNAs showed either a reduction to less than 50% or a more than twofold increase (Fig. 4a and b). Notably, most of the mRNAs found to be induced more than twofold by 0.25 μM 4SC-202 were among the low expressed genes (less than 0.5% compared to *GAPDH* mRNA), thus, bearing a higher risk of being false positive. The only two induced genes with high expression levels were *AURKA* and *PLK1* which, however, are both known to be increased during the G2/M phase [59, 60]. Hence, the observed increase may be a mere consequence of a prolonged G2/M phase in the 4SC-202 (0.25 μM)-treated cells rather than due to altered histone modification. Treatment with higher 4SC-202 concentrations (10 μM) led to gene expression changes comparable to FK228 (Fig. 4a and b) consistent with the profound alterations in H3K9ac and H3K4me2 (Additional file 1: Figs. S3a and S4).

### The 4SC-202-induced G2/M arrest of CTCL cells is independent of de novo gene expression

**Fig. 5:**
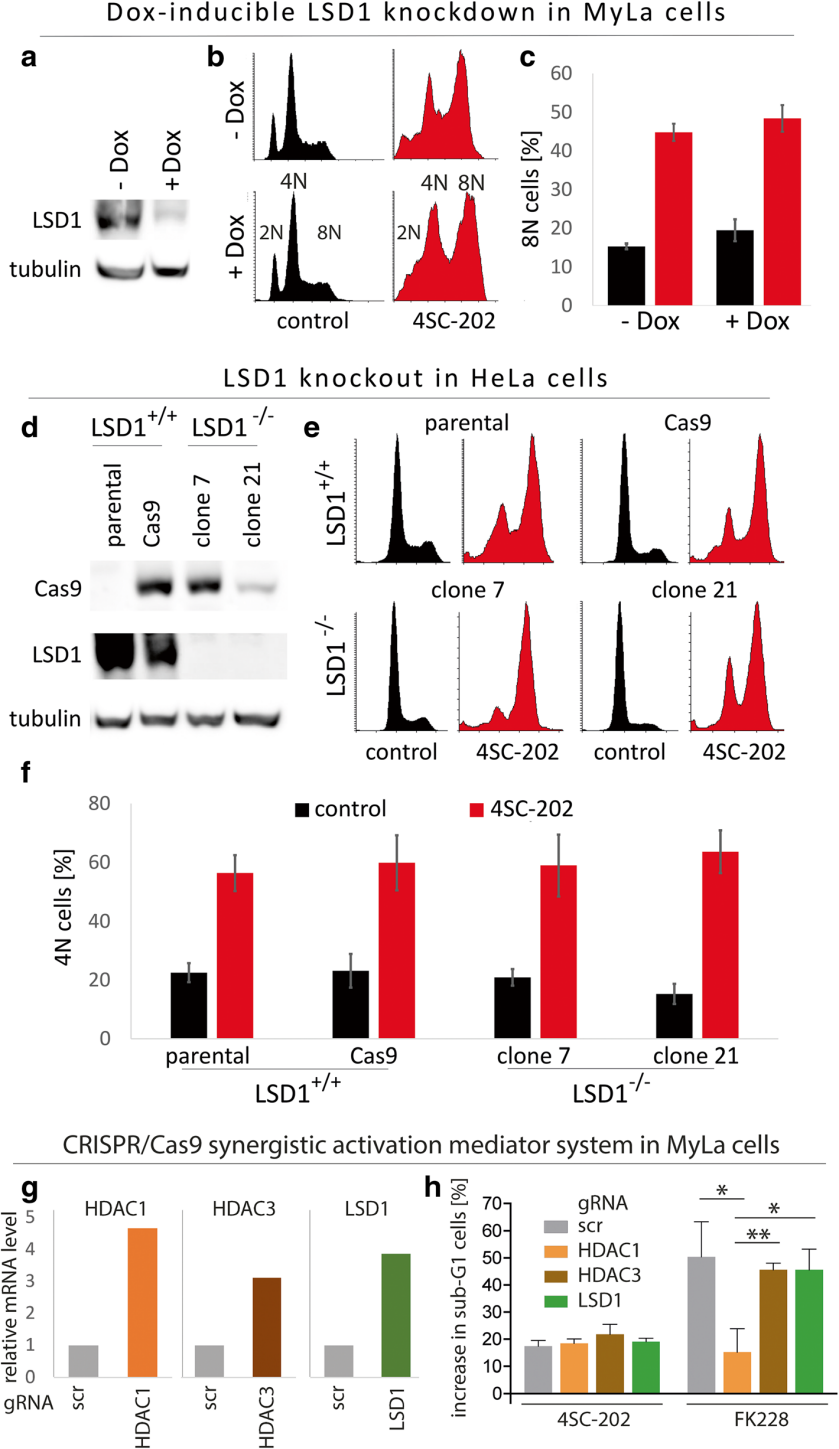
The 4SC-202-induced G2/M arrest is independent of LSD1. **a**–**c** MyLa cells were infected with a lentiviral vector allowing doxycyclin (Dox)-inducible expression of an shRNA targeting *LSD1*. Cells were treated for 5 days with 1 μg/ml Dox and **a** expression of LSD1 was assessed by immunoblot. **b** and **c** Following 5 days in the absence or presence of Dox cells were additionally treated with 1 μM 4SC-202, and cellular DNA content was analyzed after 24 h by propidium iodide staining. (Note that MyLa cells had acquired a substantial proportion of tetraploid cells following infection and selection.) **b** Representative cell cycle profiles of the partially tetraploid cells are depicted. G2/M arrest is most clearly verifiable by the increase in 8-N cells. **c** Mean values (± SD) of the percentage of 8-N cells derived from three independent experiments are given. **d**–**f** HeLa cells were infected with two lentiviral vectors allowing expression of Cas9 and a single guide RNA targeting LSD1. Two single cell clones with complete knockout of both LSD1 alleles (as confirmed by sequencing) were established and **d** lack of LSD1 expression was demonstrated by immunoblot. **e** and **f** After 24 h in the presence or absence of 1 μM 4SC-202 cellular DNA content was analyzed by propidium iodide staining. **e** Representative cell cycle profiles and **f** mean values (± SD) of the percentage of 4-N cells derived from at least five independent experiments are depicted. **g** and **h** MyLa cells were engineered to express dCas-VP64 (inactivated Cas9 fused to the VP64 transcriptional transactivator domain) and the activation helper protein MS2-p65-HSF1. To achieve specific gene activation these cells were transduced with lentiviral vectors coding for guideRNAs targeting either the *HDAC1*, the *HDAC3*, or the *LSD1* promoter. A scrambled (src) guideRNA served as control. **g** Expression levels of the indicated mRNAs were determined by SybrGreen real time PCR. **h** MyLa cells expressing the indicated guideRNA were treated with either 0.3 μM 4SC-202 or 2 nM FK228 for 48 h. Then cellular DNA content was analyzed by propidium iodide staining, and the increase of sub-G1 cells compared to the respective untreated control cells was determined. Mean values (± SD) of three independent experiments are displayed. Paired *t* test was performed (*p < 0.05; **p < 0.01)

The results so far suggested that the 4SC-202-mediated biological effects may occur independently from altered histone modifications and gene expression. To further test whether the capacity of this compound to arrest cells in the G2/M phase of the cell cycle is dependent on gene induction, we used the potent transcription inhibitor actinomycin D, which at 1 μg/ml, is completely blocking mRNA expression ([61]; Additional file 1: Figure S6a). Notably, the progress from G2/M to G1 is independent of transcription since upon release from a nocodazole-induced G2/M arrest, cells proceed to G1 irrespective of the presence of actinomycin D (Fig. 5d and Additional file 1: Figure S5 and S7). Incubation with 4SC-202, however, sustained the nocodazole-induced G2/M arrest both in the absence as well as in the presence of actinomycin D in CTCL cell lines (Fig. 5d and Additional file 1: Fig. S7a and b) and in 293 T cells (Additional file 1: Figure S6b and c). These findings indicate that 4SC-202 is able to arrest cells in G2/M without inducing gene expression. Nocodazole activates the spindle assembly checkpoint and arrests cells in prometaphase [62]. Hence, since 4SC-202 is able to maintain the nocodazole-induced G2/M arrest, 4SC-202 has to exert its function at the same point or later in mitosis. In addition, since even complete repression of transcription by actinomycin D in these cell cycle phases does not prevent the transition from prometaphase to G1, it can be concluded that for maintenance of the G2/M, arrest by 4SC-202 also gene repression is not required.

### The 4SC-202-induced G2/M arrest is independent of LSD1

Based on the inhibitory features described for FK228 [55, 56] and 4SC-202 [57], and the observation that combined siRNA-mediated knockdown of HDAC1 and HDAC3 did not induce a G2/M arrest (Additional file 1: Figure S8), the most likely explanation for the differential cellular responses induced by the two compounds would be the additional targeting of LSD1 by 4SC-202. However, as described above, our results did not suggest that the G2/M arrest induced by 4SC-202 is due to targeting a transcriptional repressor. Nevertheless, because only indirect evidence argued against LSD1 and due to the versatile functions of LSD1, which can act also as a transcriptional activator [22–24] or may modulate also the function of non-histone proteins [26], LSD1 could not be definitely ruled out as the critical target for the 4SC-202-induced G2/M arrest. Therefore, to explore further a possible role of LSD1 as 4SC-202 target, we used a Doxycyclin (Dox)-inducible shRNA targeting LSD1 to knockdown LSD1 in MyLa cells (Fig. 5a). Dox-induced reduction of LSD1 expression was neither associated with a change in cell cycle distribution of untreated cells nor was the G2/M arrest upon 4SC-202 treatment affected by reduced LSD1 levels (Fig. 5b and c). Both results argue against LSD1 being the target mediating this 4SC-202 effect.

To exclude that the residual LSD1 expression after knockdown (Fig. 5a) was still too high to significantly interfere with its function, we aimed to knockout LSD1 by CRISPR/Cas9. Due to the relatively poor infectibility of the CTCL cell lines, we did not succeed to establish CTCL cells completely devoid of LSD1 expression. Therefore, we used the cervix carcinoma cell line HeLa as the model system, which like CTCL cells undergoes G2/M arrest followed by cell death upon treatment with 4SC-202 (Additional file 1: Figure S9). Two HeLa single-cell clones completely lacking LSD1 expression were established (Fig. 5d). As in MyLa LSD1-knockdown cells, neither cell cycle distribution of untreated cells nor the G2/M arrest upon 4SC-202 treatment was affected by the absence of LSD1. Further analysis of the Sub-G1 population after 72 h demonstrated that also the induction of cell death by 4SC-202 is not affected by knockdown or knockout of LSD1 (Additional file 1: Figure S10). Thus, functional inactivation of LSD1 by 4SC-202 is neither sufficient nor required for the observed biological effects.

### Enforced expression of HDAC1 counteracts the effect of FK228 but not of 4SC-202

Recently, targeting of class I HDACs by 4SC-202 has been proposed as the critical mechanism for inhibiting oncogenic signaling and proliferation in medulloblastoma cells [63]. To further sustain our results arguing against the involvement of HDAC for 4SC-202 activity in CTCL cell lines, we aimed to directly test the impact of class I HDACs on the response towards 4SC-202 and investigated for comparison also the class I HDAC inhibitor FK228. Real time PCR analysis had demonstrated that out of the HDACs targeted by both drugs, *HDAC1* and *3* are predominant in CTCL cells (Additional file 1: Figure S1). We, therefore, applied the CRISPR/Cas9 synergistic activation mediator (SAM) system [64] to further increase expression of HDAC1, HDAC3, and for control purposes LSD1 in MyLa cells (Fig. 5g and Additional file 1: Figure S11a and b). In line with class I HDACs being the critical targets, cell death induced by 2 nM FK228 was significantly reduced in MyLa cells expressing elevated levels of HDAC1 (Fig. 5h). In contrast, no reduction of induced cell death was observed in case of treatment with 0.3 μM 4SC-202 (Fig. 5h).

### 4SC-202 induces major defects in the process of mitosis

**Fig. 6:**
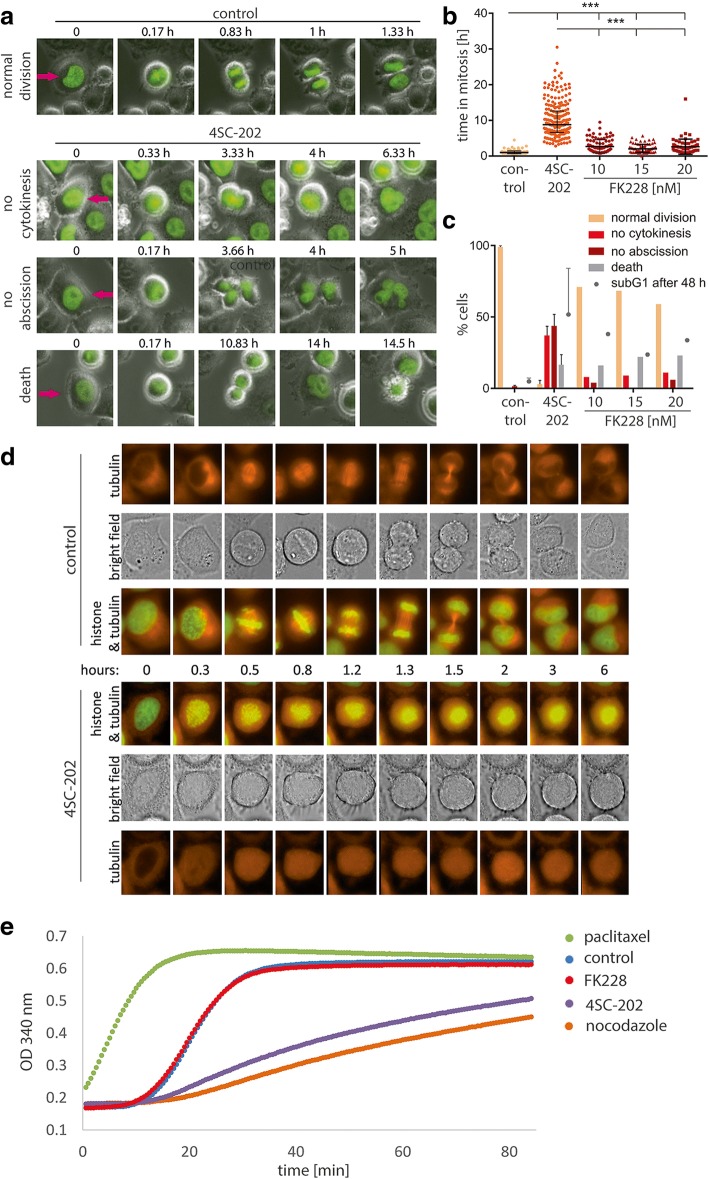
4SC-202 delays mitosis, interferes with spindle formation, and can directly affect tubulin polymerization. **a**–**c** HeLa cells expressing a histone H2B-GFP fusion protein were treated with 1 μM 4SC-202 and the indicated FK228 concentrations and analyzed by time-lapse microscopy for 48 h. Detachment and rounding up of the previously adherent cells was regarded as onset of mitosis. **a** Photo series illustrating typical outcomes following detachment in the absence or presence of 4SC-202. **b** Time in mitosis was recorded as the duration of detachment of individual cells. The plot depicts singular value distribution, their median and the interquartile ranges. Statistical significance was tested using nonparametric Kruskal-Wallis test followed by Dunn’s multiple comparison post test (***p < 0.001). **c** The frequency of the events depicted in sub-figure a) was recorded by counting cells (between 56 and 253 events) for the indicated three different FK228 concentrations (each *n* = 1) and in three independent experiments for 1 μM 4SC (mean values ± SD). Additionally the percentage of sub-G1 cells was determined following propidium iodide staining. **d** Photo series derived from time-lapse microscopy of HeLa cells expressing a red fluorescent tubulin-RFP in addition to the H2B-GFP fusion protein. Cells were cultured in the absence or presence of 1 μM 4SC-202. **e** An in vitro tubulin polymerization assay was performed by addition of GTP to a > 99% pure bovine tubulin solution and monitoring of microtubule formation by measuring the OD at 340 nm in the course of time. All indicated substances were added at a concentration of 10 μM as suggested by the manual

All results so far argued for 4SC-202 targeting molecules beyond HDACs and LSD1, and the most obvious biological difference compared to FK228 was the induction of a G2/M arrest. To determine how 4SC-202 affects mitosis, we applied time-lapse microscopy. Because this is not feasible with CTCL cells, which are growing as spheroids in suspension, we used again HeLa cells as the model system. To facilitate time-lapse microscopic observation of mitosis, we used HeLa cells expressing a green fluorescent H2B-GFP fusion protein. Normal cell division in untreated control cells was accomplished within 1 h (Fig. 6a). In the presence of 4SC-202, however, this process was significantly disturbed: cells detached and rounded up normally at the onset of mitosis but in contrast to control cells remained in this state for 8.8 h on average (Fig. 6a and c). The delayed detachment phase was then ceased by an aberrant cell division. In 37% of the quantified cells, no nuclear division occurred, and cells settled down again without having divided (Fig. 6a and b). Lack of abscission and generation of di-nuclear cells manifested in more than 40% while cell death directly following aberrant mitosis was observed in 17% of the analyzed cells (Fig. 6b). In contrast to these major defects in mitosis conveyed by 4SC-202 treatment, FK228 applied at different concentrations resulted in only a slight delay of the mitotic process (Fig. 6b) and in only slightly increased failure of cytokinesis or abscission (Fig. 6c).

### 4SC-202 inhibits tubulin polymerization

To further visualize the mitotic perturbations induced by 4SC-202, we made use of H2B-GFP HeLa cells additionally expressing tubulin-RFP. Here, it became evident that condensation of the chromosomes and breakdown of the nuclear lamina (evident by tubulin being evenly distributed throughout the cell) occurred normally in the 4SC-202-treated cells (Fig. 6d). Formation of the mitotic spindle, however, and subsequent formation of metaphase plate were impaired (Fig. 6d) suggesting that 4SC-202 directly or indirectly might act as a “spindle poison”. To test whether 4SC-202 is directly affecting tubulin polymerization, we applied an in vitro tubulin polymerization assay, which allows assessing polymer formation in a tubulin solution by monitoring the OD at 340 nm over time. While paclitaxel, a molecule known to stabilize microtubules, accelerated the process, 4SC-202, like nocodazole—a known inhibitor of tubulin polymerization—decreased the polymerization rate (Fig. 6e). In contrast, FK228 did not affect in vitro microtubule formation. To confirm that 4SC-202 affects also intracellular tubulin polymerization, we applied an assay allowing quantification of intracellular tubulin polymers [54]. Indeed, we could demonstrate in HeLa, as well as in two CTCL cell lines (HTB-176 and CRL-2105) that 4SC-202 reduces intracellular polymerized tubulin to a similar degree as nocodazole (Additional file 1: Fig. S12a). In a second set of experiments, we exploited the fact that activation of the spindle assembly checkpoint (SAC) is essential for mitotic arrest induced by drugs affecting spindle formation [65]. The key SAC activating kinase is monopolar spindle 1 (MPS1), and a characteristic feature of a microtubule defect-induced cell cycle arrest is that it can be overcome by inhibiting MPS1 [66, 67]. Indeed, the specific MPS1-inhibitor BAY1217389 induces in cells arrested by nocodazole as well as by 4SC-202 a premature exit from mitosis, as indicated by a reduction of Cyclin B1 expression and reattachment of detached cells (Additional file 1: Fig. S12b and c).

## Discussion

Recent pre-clinical studies on 4SC-202 have demonstrated activity against hepatocellular carcinoma [68], colorectal cancer [69], urothelial carcinoma [57], medulloblastoma [63, 70], and pancreatic cancer cells [71]. Our study adds cutaneous T cell lymphoma to this list. Moreover, in line with previous publications [69, 70], we observed that cytotoxicity induced by 4SC-202 was elevated in cancer cells compared to untransformed control cells.

Interestingly, different mechanisms have been proposed for how 4SC-202 would inhibit cellular growth and viability. In this respect, activation of the ASK1-dependent mitochondrial apoptosis pathway in hepatocellular carcinoma cells [68], inhibition of hedgehog/GLI signaling in medulloblastoma cells [63], or increased expression of BRD4- and MYC-dependent epithelial genes in pancreatic cancer cells [71] have been suggested as critical anti-tumorigenic outcome following inhibition of its primary targets by 4SC-202. Indeed, all previous studies on 4SC-202 have in common that the authors either assume or provide evidence that these primary targets are class I HDACs and/or LSD1. In contrast, our results suggest that 4SC-202 has the potential to inhibit cancer cell growth also independent of targeting these epigenetic modifiers, and independent of altering cellular transcription.

Our study on CTCL cells was largely based on the comparison of 4SC-202 with FK228, a class I HDAC inhibitor approved in the US for the treatment of CTCL [72, 73]. One major difference regarding the biological effects induced by the two inhibitors was that cell death induction by 4SC-202 was preceded by a G2/M arrest, a feature of 4SC-202 reported by others as well [57, 69]. Since HDACs1–3 are also inhibited by FK228 [55, 56], the only other described target of 4SC-202, i.e., LSD-1, [43] seemed to be the best candidate to mediate the observed G2/M arrest. However, neither knockdown nor knockout of LSD-1 affected cell cycle distribution of untreated or 4SC-202-treated cells. Furthermore, and in contrast to FK228, 4SC-202-induced cytotoxicity could not be attenuated by HDAC1 overexpression, and was observed already at concentrations associated with only minor changes in histone modifications and gene transcription. Finally, even under conditions of completely abolished cellular transcription, 4SC-202 could maintain a G2/M arrest. Together, these results suggest that 4SC-202 can induce mitotic arrest and cell death by targeting molecules which differ from its targets described so far. Our data, however, do not suggest that 4SC-202 cannot inhibit HDACs and LSD-1 in CTCL or other cells. Nevertheless, it is of interest that a recent publication raised doubts whether 4SC-202 would target LSD1 at all [40].

The major structural elements of the mitotic spindle are microtubule polymers consisting of α/β tubulin heterodimers. [74] Spindle assembly as well as chromosome segregation during mitosis are highly complex processes involving multiple accessory proteins [74, 75]. Nevertheless, spindle formation and function are based on the fundamental capability of tubulin to undergo spontaneous microtubule polymerization in the presence of GTP [76]. Disturbing microtubule polymerization has proven efficacy in the clinic against a broad range of malignancies [77], and respective antimitotic drugs are usually classified as either microtubule-destabilizing agents, which inhibit microtubule polymerization (e.g., nocodazole) or microtubule-stabilizing agents (e.g., paclitaxel) [74]. In a very simple in vitro system consisting of highly purified α- and β-tubulin and GTP, we demonstrated here that 4SC-202 is able to inhibit tubulin polymerization just like nocodazole suggesting that it might act as a microtubule-destabilizing spindle poison. Indeed, using time-lapse microscopy, we could confirm that 4SC-202 is able to inhibit formation of the mitotic spindle in HeLa cells. Since 4SC-202-treated CTCL cells display a reduction of polymerized tubulin in 4-N cells as well as activation of the spindle assembly checkpoint, it is very likely that also in CTCL cells 4SC-202 is affecting formation of the mitotic spindle and thereby induces the observed G2/M arrest. Furthermore, since a 4SC-202-induced mitotic arrest has been described for colorectal cancer [69] and urothelial carcinoma cells [57], and we observed the same in six out of six cell lines from further cancer entities, targeting of tubulin polymerization might be a general mechanism contributing to impairment of tumor cells by 4SC-202. This probably includes also cytotoxic effects induced by 4SC-202 since it is well known that inhibiting mitosis finally leads to cell death [77]. Because it is indicating a potentially large therapeutic window, it is of special interest that arrest and cell death induced by 4SC-202 occurred only in cancer cells. Indeed, even fast-proliferating control cells were barely affected by 4SC-202 treatment. Since it has been shown that modifying microtubule stability can change the sensitivity towards nocodazole [78], a possible explanation for the differential sensitivity towards 4SC-202 might be diverse microtubule stability in cancer and control cells.

Emerging therapies for leukemia and lymphoma are frequently based on the application of epigenetic modifiers and especially HDAC inhibitors [79–81]. Our data suggest that in contrast to most other HDAC inhibitors, 4SC-202 is additionally directly targeting microtubule formation. Polypharmacology, a concept encompassing both, multiple drugs binding different targets as well as one drug binding multiple targets within a pathological network, is considered as a very useful strategy for the treatment of complex and refractory diseases, in particular of cancer [82]. In this respect, attempts have been made to design new drugs targeting HDACs as well as tubulin polymerization, and recently such compounds demonstrating excellent anti-proliferative activity have been described [83]. Furthermore, mocetinostat, an inhibitor of class I/IV HDACs which is currently under clinical evaluation [84], has been demonstrated to also bear microtubule inhibitory activity although direct interaction with tubulin was not formally proven [85]. Notably, HDAC inhibitors can disrupt mitosis by affecting a number of components of the mitotic machinery [86]. Our results, however, argue against such an indirect effect. Indeed, here we describe that 4SC-202, which so far was considered as a drug targeting class I HDACs as well as the histone demethylase LSD1, is also a potent microtubule-destabilizing agent. The latter function was dominant for the impairment of CTCL cell lines in our in vitro experiments. This, however, does not exclude a contribution of the other inhibitory features to the anti-tumoral activity of the substance. This holds true for direct effects on cancer cells but even more for indirect effects, which cannot be detected in vitro. In this respect, it is intriguing that very recently LSD1 was demonstrated to be critical for inhibiting tumor cell immunogenicity [87]. Furthermore, also HDAC inhibitors are discussed as potential immunomodulating agents to treat cancer [88]. Therefore, it will be interesting to evaluate whether the pleiotropic molecular features of 4SC-202 may turn into benefits for treated cancer patients. In this respect, a first-in-man phase I clinical trial with 4SC-202 (www.clinicaltrials.gov, NCT01344707) for patients with advanced hematological malignancies has been launched [44] and a report of the results is awaited soon.

## Conclusions

We provide pre-clinical in vitro data that cutaneous T cell lymphoma cells are specifically sensitive towards 4SC-202, a compound previously described as a dual inhibitor targeting type I HDACs as well as LSD1. However, our experiments suggest that 4SC-202-induced G2/M arrest and cell death is not due to targeting these epigenetic modifiers, but that 4SC-202 has a further molecular function by directly inhibiting tubulin polymerization.

## Additional file

Additional file 1: **Table S1**. Doubling time in hours of the investigated cell lines and fibroblasts B. **Table S2**. Real time PCR primers. **Figure S1**. mRNA expression levels of the four 4SC-202 targets. Fig. S2. 4SC-202 induces G2/M arrest in cell lines from different cancer entities. **Figure S3.** The G2/M arrest induced by different concentrations of 4SC-202 inversely correlates with the level of histone modifications. **Figure S4.** Concentration dependent induction of histone modifications by 4SC-202. **Figure S5.** G2/M arrest and cell death induced by 4SC-202 and FK228 concentrations applied in the NanoString experiment (Fig. 4a and b). **Figure S6.** Inhibition of gene transcription by 1 μg/ml actinomycin D. **Figure S7.** Active gene transcription is not required for a 4SC-202-mediated G2/M arrest. **Figure S8.** HDAC1/HDAC3 double knockdown does not lead to induction of a G2/M arrest. **Figure S9.** 4SC-202 induces cell death preceded by a G2/M arrest in HeLa cells. **Figure S10.** LSD1 knockdown or knockout does not affect cell death induced by 4SC-202. **Figure S11.** Enforced expression of HDAC1 counteracts FK228 but not 4SC-202. **Figure S12.** 4SC-202 reduces the fraction of intracellular polymeric tubulin and activates the spindle assembly checkpoint. (PDF 2399 kb)

## Abbreviations

CTCL: Cutaneous T cell lymphoma
HDAC: Histone deacetylase
LSD1: Lysine-specific demethylase 1
