# Supplementary material for: Elucidating the mechanism of action of domatinostat (4SC-202) in cutaneous T cell lymphoma cells
Source: J Hematol Oncol. 2019 Mar 18;12:30. doi: 10.1186/s13045-019-0719-4 (PMC6423872; doi:10.1186/s13045-019-0719-4)
Supplement: Supplementary file 1 — Table S1. Doubling time in hours of the investigated cell lines and fibroblasts B. Table S2. Real time PCR primers. Figure S1. mRNA expression levels of the four 4SC-202 targets. Fig. S2. 4SC-202 induces G2/M arrest in cell lines from different cancer entities. Figure S3. The G2/M arrest induced by different concentrations of 4SC-202 inversely correlates with the level of histone modifications. Figure S4. Concentration dependent induction of histone modifications by 4SC-202. Figure S5. G2/M arrest and cell death induced by 4SC-202 and FK228 concentrations applied in the NanoString experiment (Fig. 4a and b). Figure S6. Inhibition of gene transcription by 1 μg/ml actinomycin D. Figure S7. Active gene transcription is not required for a 4SC-202-mediated G2/M arrest. Figure S8. HDAC1/HDAC3 double knockdown does not lead to induction of a G2/M arrest. Figure S9. 4SC-202 induces cell death preceded by a G2/M arrest in HeLa cells. Figure S10. LSD1 knockdown or knockout does not affect cell death induced by 4SC-202. Figure S11. Enforced expression of HDAC1 counteracts FK228 but not 4SC-202. Figure S12. 4SC-202 reduces the fraction of intracellular polymeric tubulin and activates the spindle assembly checkpoint. (PDF 2399 kb) [file 13045_2019_719_MOESM1_ESM.pdf]

Supplementary table 1: Doubling time in hours of the investigated cell lines and fibroblasts B

CRL-2105	CRL-8294	HTB-176	HuT 78	MyLa	Se-Ax	fibroblasts B	HeLa
40	44	22	21	29	34	30	24

Supplementary table 2: Real time PCR primers

target gene	forward	reverse
RPLPO	CCA TCA GCA CCA CAG CCT TC	GGC GAC CTG GAA GTC CAA CT
LSD1	AAT GCC AAA GCA GAG AAG GA	GTC ATC CGG TCA TGA GGA AG
HDAC1	CAC GGA CCG GGT CAT GAC TGT	CTT GCC TTT GCC AGC CCC GA
HDAC2	TCA AGG AGG CGG CAA AAA	TGC GGA TTC TAT GAG GCT TCA
HDAC3	CTG TGT AAC GCG AGC AGA AC	GCA AGG CTT CAC CAA GAG TC

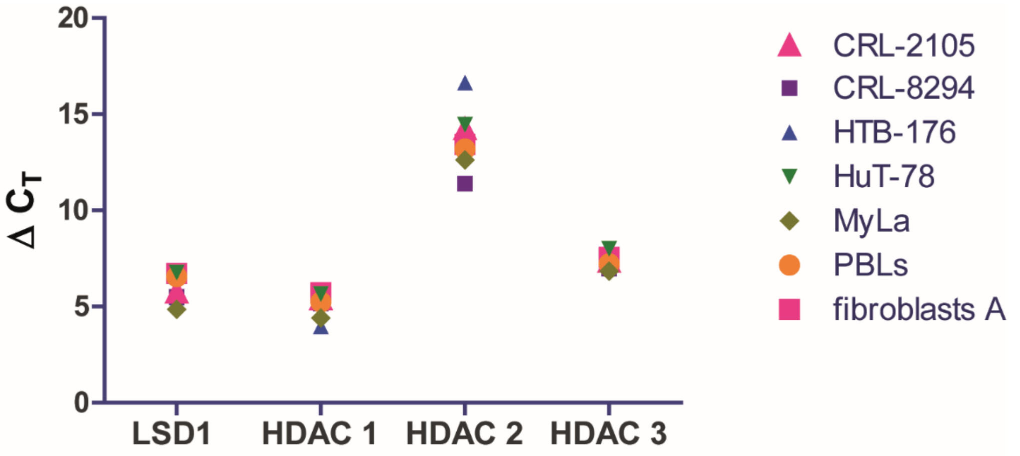

Supplementary Figure S1: mRNA expression levels of the four 4SC-202 targets. Total RNA was isolated from the indicated cells and reverse transcribed into cDNA. SybrGreen real time PCR was performed with primers for the indicated genes as well as for RPLP0 and ΔC_T values were determined.

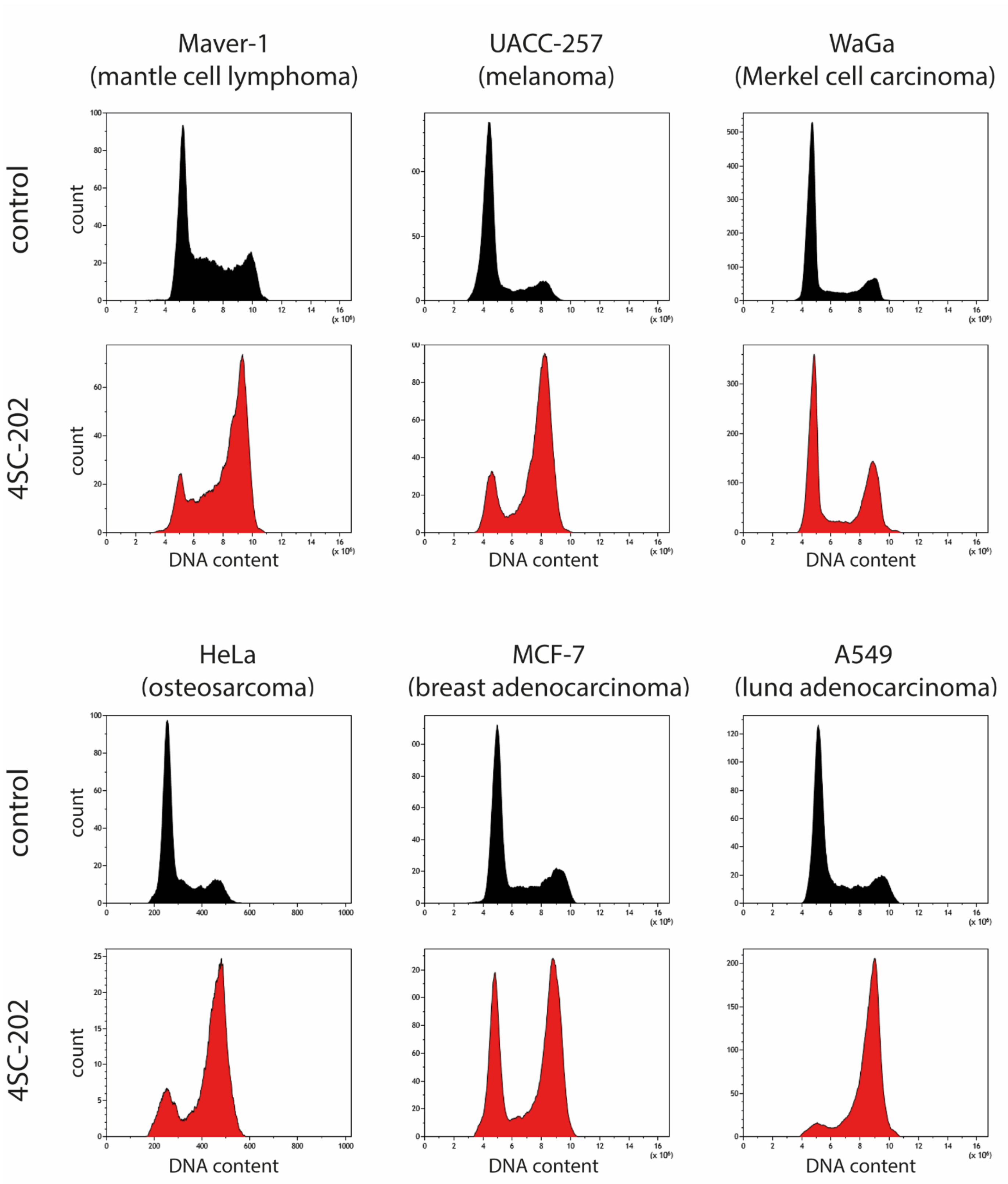

Supplementary Figure S2: 4SC-202 induces G2/M arrest in cell lines from different cancer entities. Following 24 hours of incubation with 2 µM 4SC-202, the indicated cell lines were fixed, DNA was stained with propidium iodide and analyzed by flow cytometry. Gating was performed to display only cells with 2N to 4N DNA content. Note, that WaGa (doubling time: 3 days) is a very slowly proliferating cell lines, presumably accounting for the relatively minor accumulation of 4N cells.

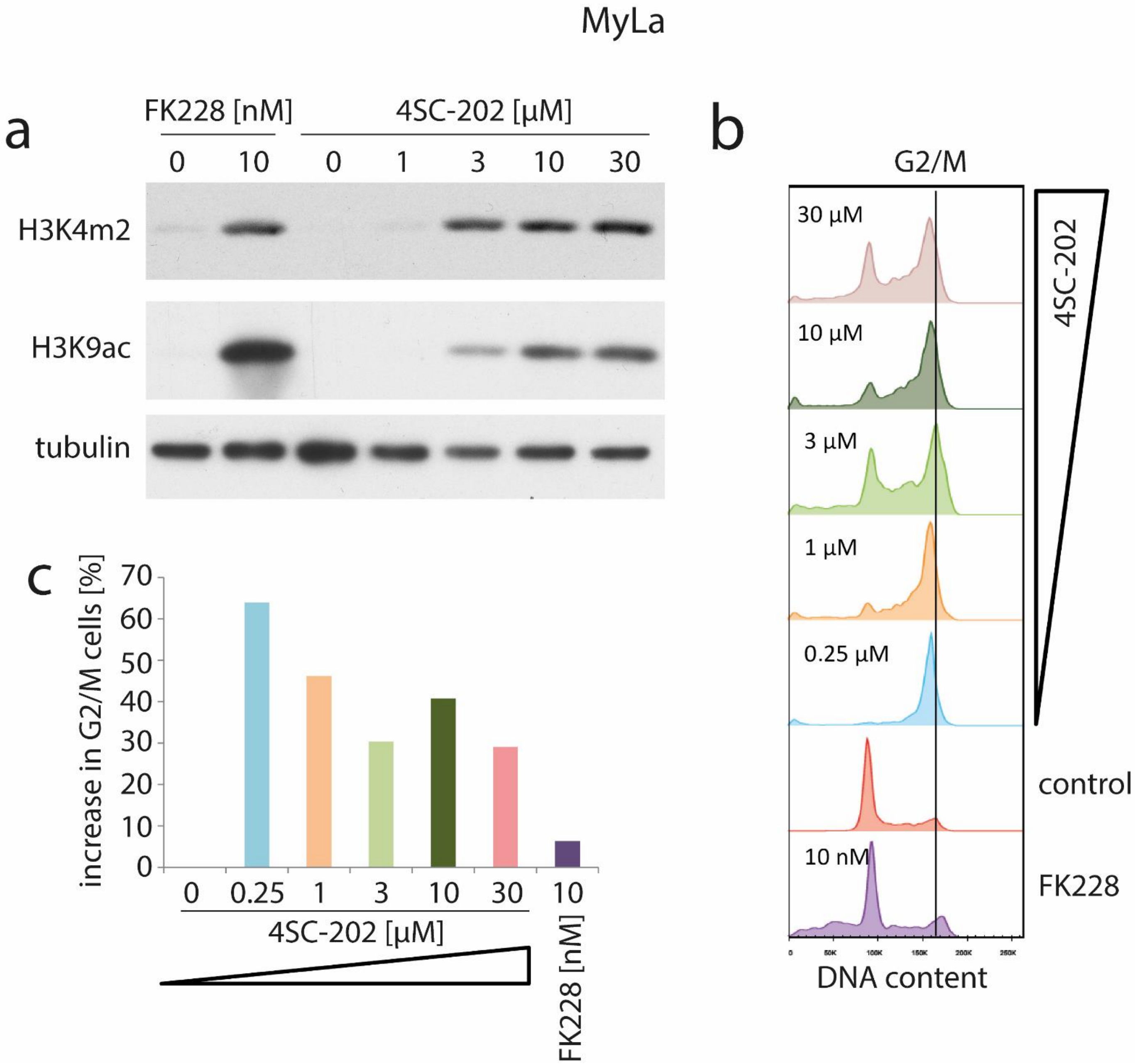

Supplementary Figure S3: The G2/M arrest induced by different concentrations of 4SC-202 inversely correlates with the level of histone modifications. MyLa cells were treated with the indicated drug concentrations for 24 hours. **a)** Total cell lysates were subjected to immunoblot analysis using antibodies recognizing histone H3 dimethyl lysine 4 (H3K4me2) or histone H3 acetyl lysine 9 (H3K9ac). Tubulin served as loading control. **b) and c)** Cellular DNA content was analyzed by PI staining of fixed cells. **b**) Cell cycle profiles are given. **c)** The increase in G2/M cells relative to untreated cells is displayed.

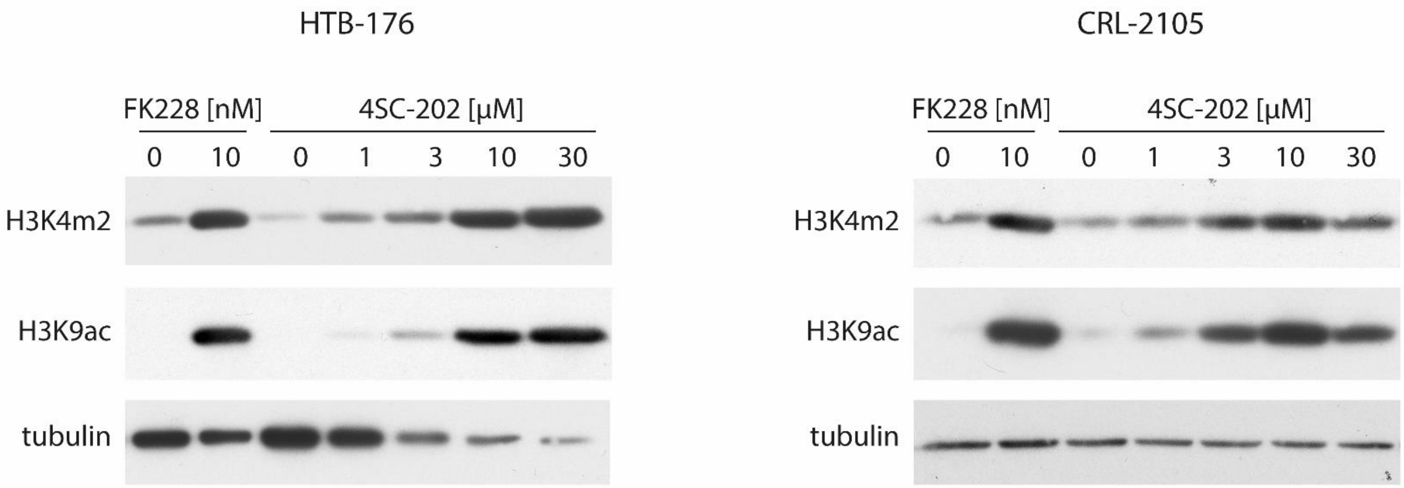

Supplementary Figure S4: Concentration dependent induction of histone modifications by 4SC-202. HTB-176 and CRL-2105 cells were treated with the indicated drug concentrations. Total cell lysates harvested after 24 hours of drug treatment were subjected to immunoblot analysis using antibodies recognizing histone H3 dimethyl lysine 4 (H3K4me2) or histone H3 acetyl lysine 9 (H3K9ac). Tubulin served as loading control.

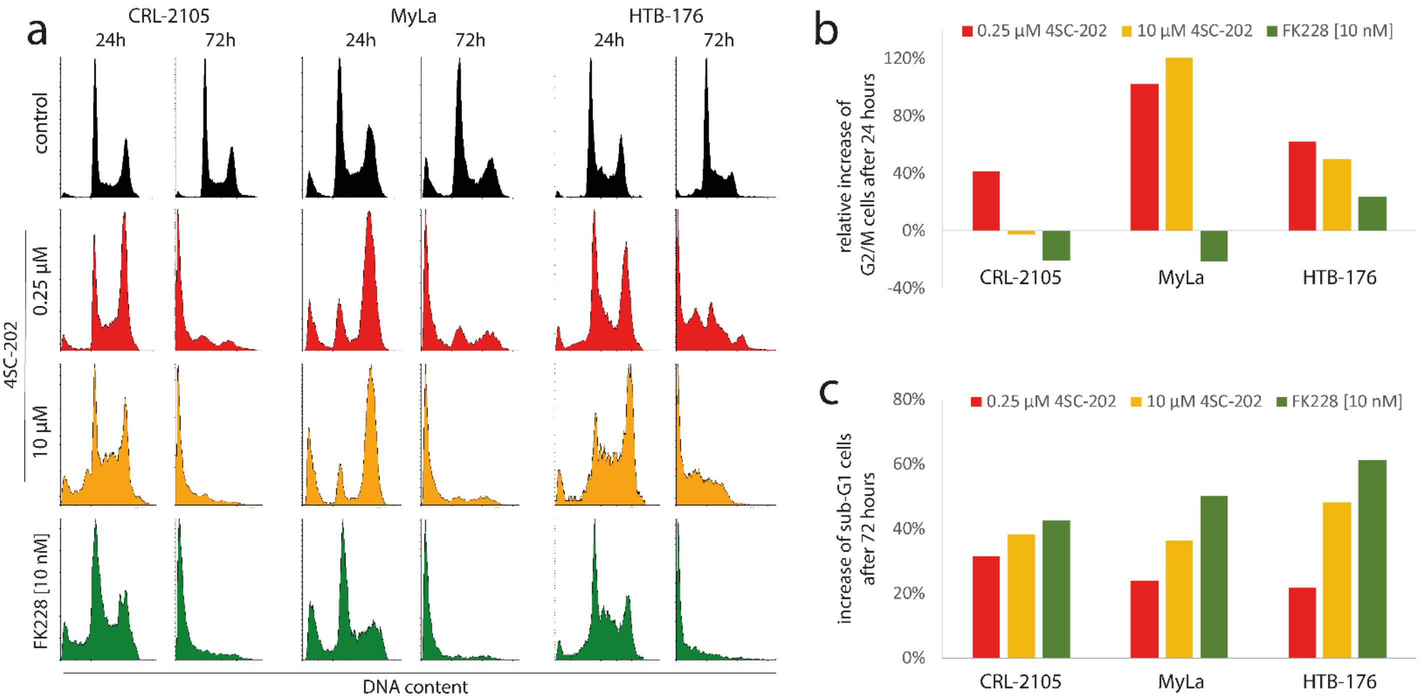

Supplementary Figure S5: G2/M arrest and cell death induced by 4SC-202 and FK228 concentrations applied in the NanoString experiment (Figure 4a and b). The indicated cell lines were incubated with 4SC-202 (0.25 µM and 10 µM) or FK228 (10 nM) for 24 or 72 hours. Then genomic DNA of fixed cells was stained with propidium iodide and analyzed by flow cytometry **a)** Histograms of cellular DNA content are depicted. **b)** The increase of cells with 4N DNA content after 24 hours of drug treatment divided by the percentage of 4N cells in the control sample is given for the indicated cell lines and conditions. **c)** The increase of sub-G1 after 72 hours of drug treatment compared to the control samples is given for the indicated cell lines and conditions.

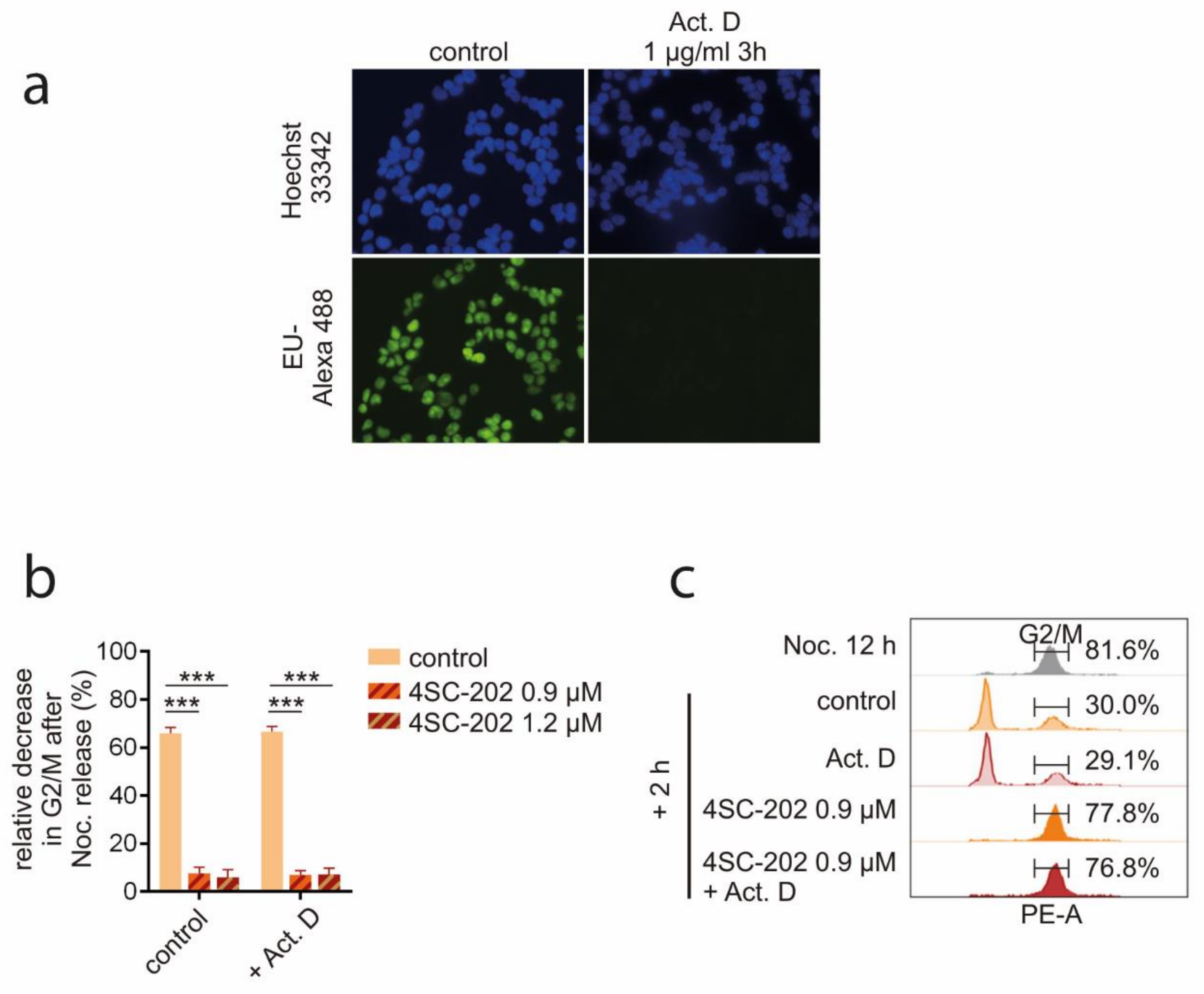

Supplementary Figure S6: Inhibition of gene transcription by 1 µg/ml actinomycin D. **a)** Inhibition of transcription by 1 µg/ml actinomycin D was confirmed in HEK293T cells by using the Click-iT RNA Alexa Fluor 488 Imaging Kit® (Thermo Fisher). The principle of this method is the incorporation of 5-Ethynyl Uridine (EU), a modified nucleotide, into newly synthesized RNA, which can then be visualized by click chemistry leading to fluorescent labeling of the RNA (EU-Alexa 488). **b) and c)** To test the impact of inhibited transcription on the ability of 4SC-202 to support a G2/M arrest in HEK293T, cells were first arrested in prometaphase by a 12 h nocodazole (100 nM) treatment. Cell cycle analysis of nocodazole-synchronized CTCL cells and subsequent treatment with 4SC-202, with and without addition of actinomycin D.

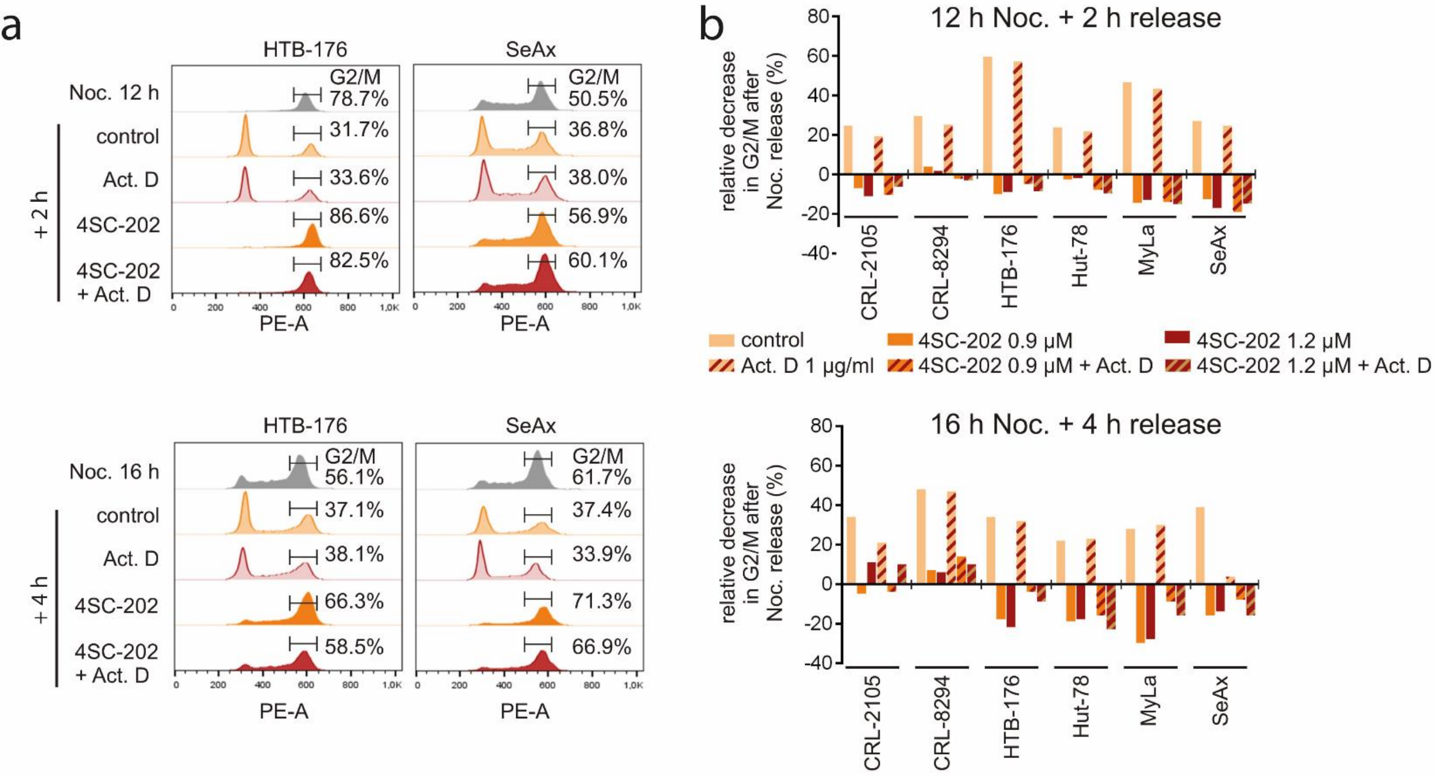

Supplementary Figure S7: Active gene transcription is not required for a 4SC-202-mediated G2/M arrest. To evaluate the necessity of *de novo* gene expression for the 4SC-202-induced G2/M arrest, the indicated cell lines were first arrested in prometaphase by a 12 or 16 h treatment with nocodazole (Noc.). Then nocodazole was removed, and cell cycle progression in the presence or absence of the transcription inhibitor Actinomycin D (Act.D) and/or 4SC-202 was assessed by propidium iodide staining. **a)** Cell cycle profiles for HTB-176 and Se-Ax are given. **b)** The decrease of G2/M cells under the different indicated conditions after removal of nocodazole is displayed .

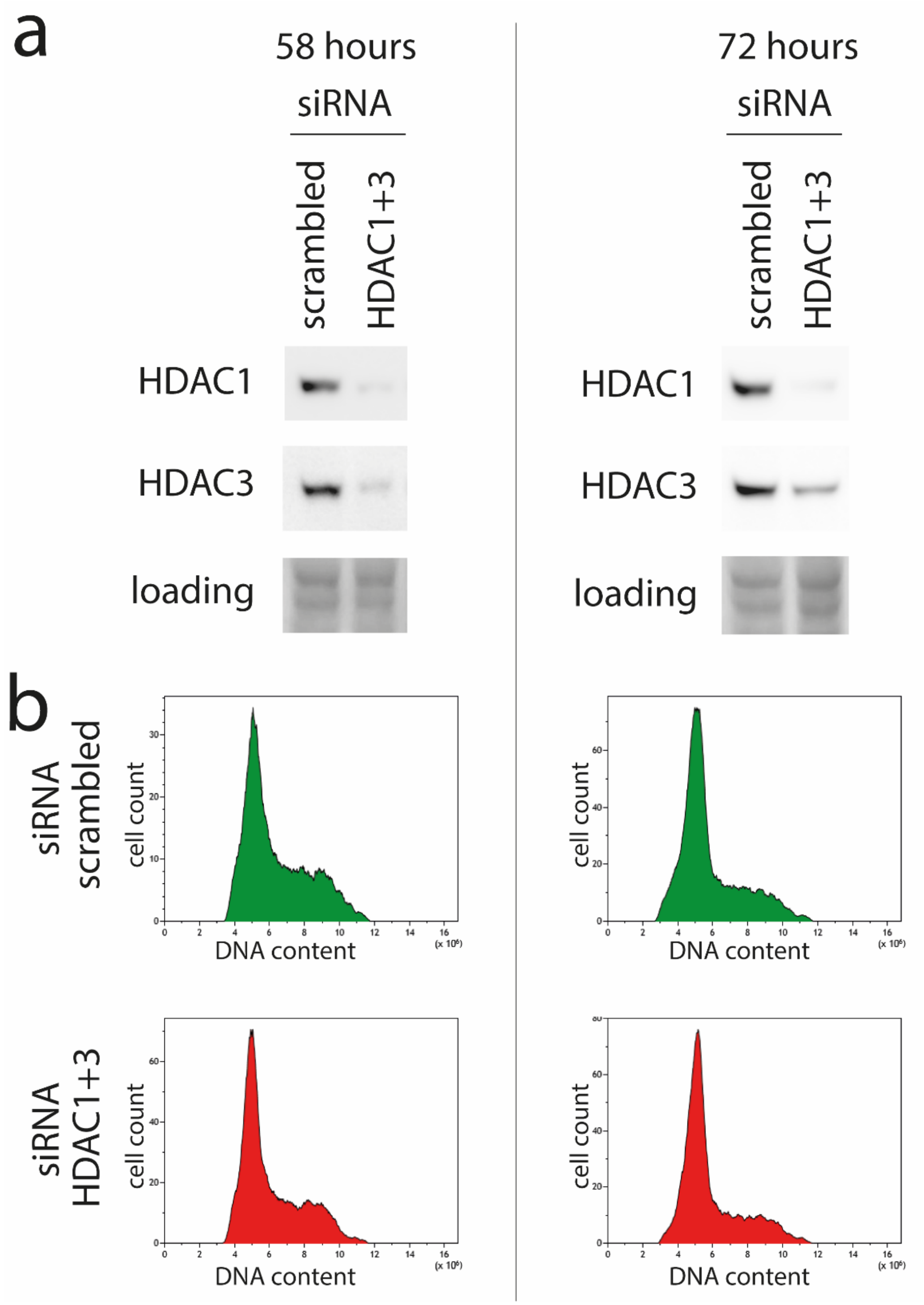

Supplementary Figure S8: HDAC1/HDAC3 double knockdown does not lead to induction of a G2/M arrest. HeLa cells were transfected either with a scrambled control siRNA or with two siRNAs targeting HDAC1 or HDAC3, respectively. At the indicated time points cells were fixed for cell cycle analysis and total cell lysates were harvested. **a)** Immunoblot analysis using antibodies recognizing HDAC1 and HDAC3. Equal loading was evaluated by PoinceauS staining. **b)** Genomic DNA of fixed HeLa cells was stained with propidium iodide and analyzed by flow cytometry. Gating was performed to display only cells with 2N to 4N DNA content

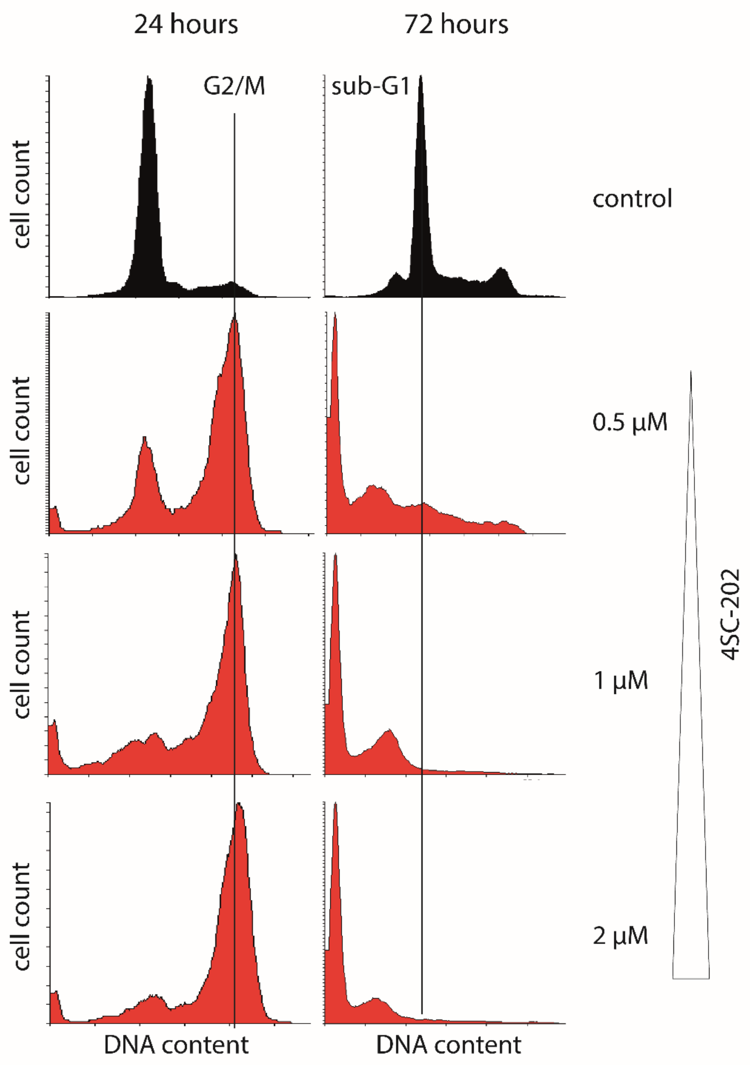

Supplementary Figure S9: 4SC-202 induces cell death preceded by a G2/M arrest in HeLa cells. Following 24 or 72 hours of incubation with the indicated concentrations of 4SC-202 genomic DNA of fixed HeLa cells was stained with propidium iodide and analyzed by flow cytometry.

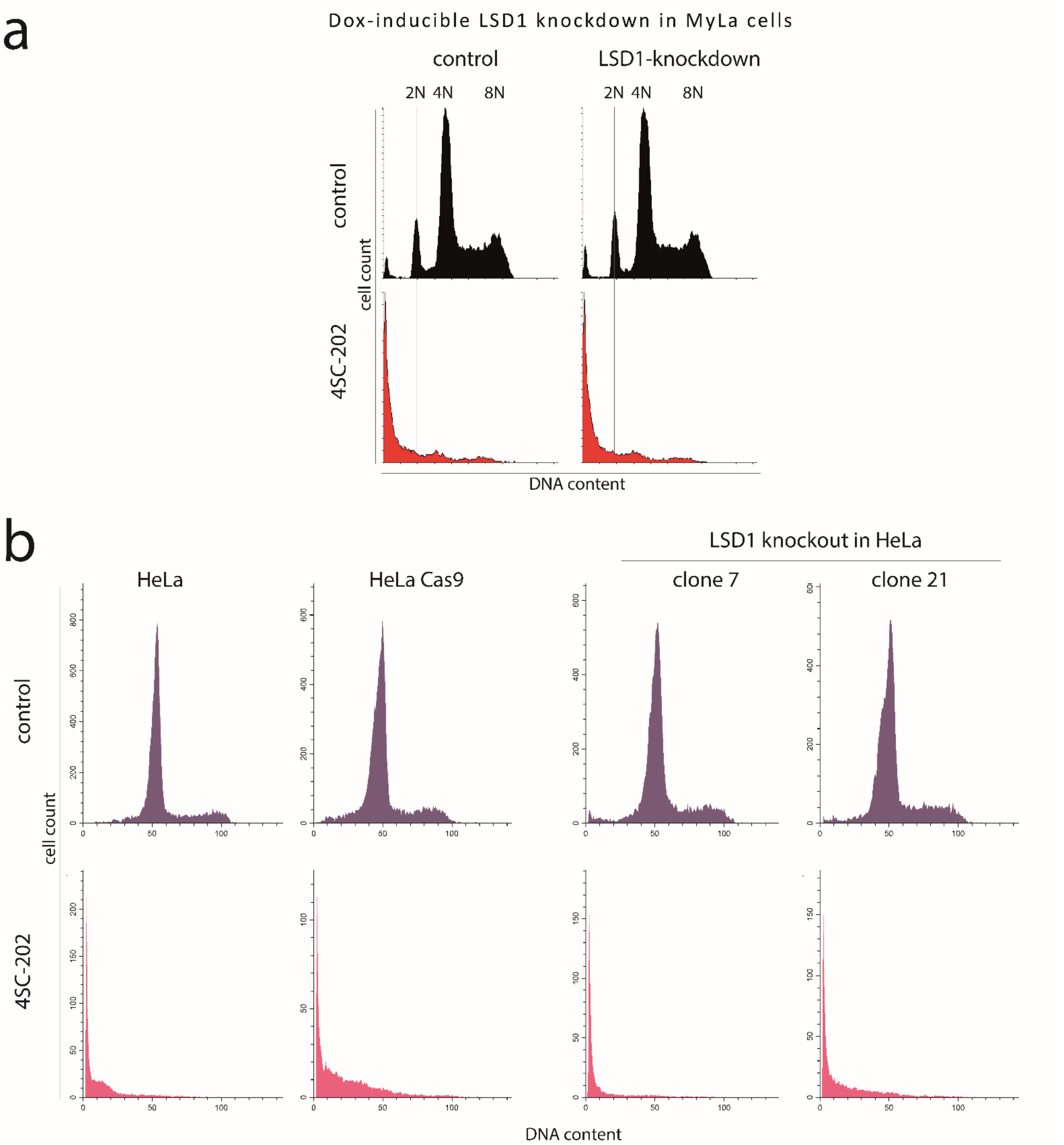

Supplementary Figure S10: LSD1 knockdown or knockout does not affect cell death induced by 4SC-202. a) MyLa cells were infected with a lentiviral vector allowing doxycyclin (Dox) inducible expression of an shRNA targeting *LSD1*. Following 5 days in the absence or presence of Dox which leads to a substantial LSD1 knockdown (Figure 5a) cells were additionally treated with 1 µM 4SC-202 and cellular DNA content was analyzed after 72 hours by propidium iodide staining. **b)** HeLa cells were infected with two lentiviral vectors allowing expression of Cas9 and a single guide RNA targeting LSD1. Two single cell clones with complete knockout of both LSD1 alleles were established and lack of LSD1 expression was confirmed by immunoblot (Figure 5 d). After 72 hours in the presence or absence of 1 µM 4SC-202 cellular DNA content was analyzed by propidium iodide staining.

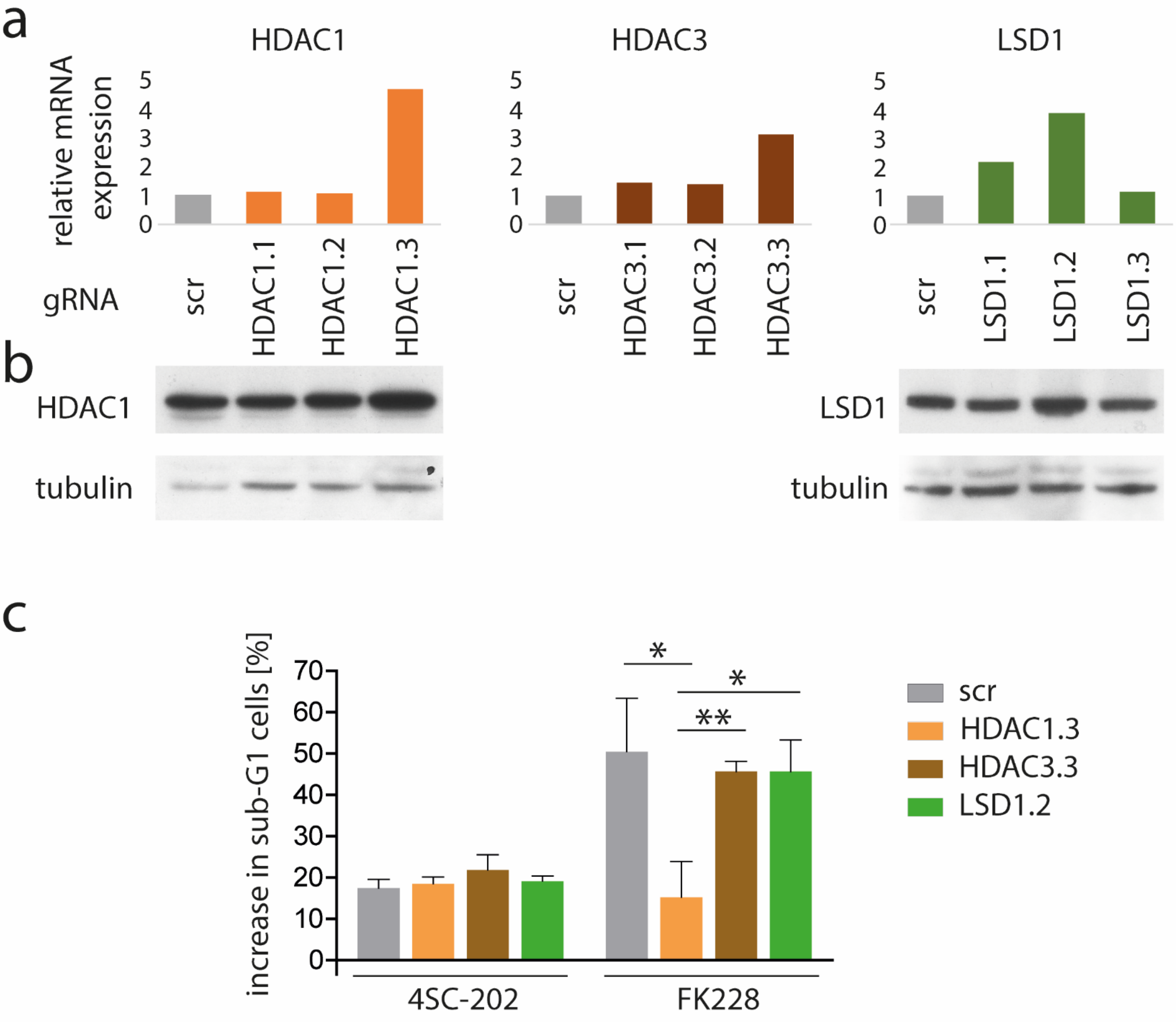

Supplementary Figure S11: Enforced expression of HDAC1 counteracts FK228 but not 4SC-202. MyLa cells were engineered to express dCas-VP64 (inactivated Cas9 fused to the VP64 transcriptional transactivator domain) and the activation helper protein MS2-p65-HSF1. **a) and b)** To achieve specific gene activation these cells were transduced with lentiviral vectors coding for different guideRNAs targeting either the *HDAC1* (HDAC1.1-3), the *HDAC3* (HDAC3.1-3) or the *LSD1* (LSD1.1-3) promoter. A scrambled (src) guideRNA served as control. **a)** Total RNA was isolated and reverse transcribed into cDNA. SybrGreen real time PCR was performed for *HDAC1* or *HDAC3* and *RPLP0* as reference. ΔC_T values were determined and relative expression calculated using the $\Delta\Delta C_T$ method and scr as calibrator. **b)** Total cell lysates were analyzed for the expression of HDAC1 by immunoblot. For HDAC3 no suitable antibody was available. **c)** MyLa cells expressing scr, HDAC1.3, HDAC3.3 or LSD1.2 guideRNA were treated with either 0.3 µM 4SC-202 or 2 nM FK228 for 48 hours. Then cellular DNA content was analyzed by propidium iodide staining, and the increase of sub-G1 cells compared to the respective untreated control cells was determined. Mean values (+SD) of three independent experiments are displayed. Paired t test was performed (* p<0.05; ** p <0.01).

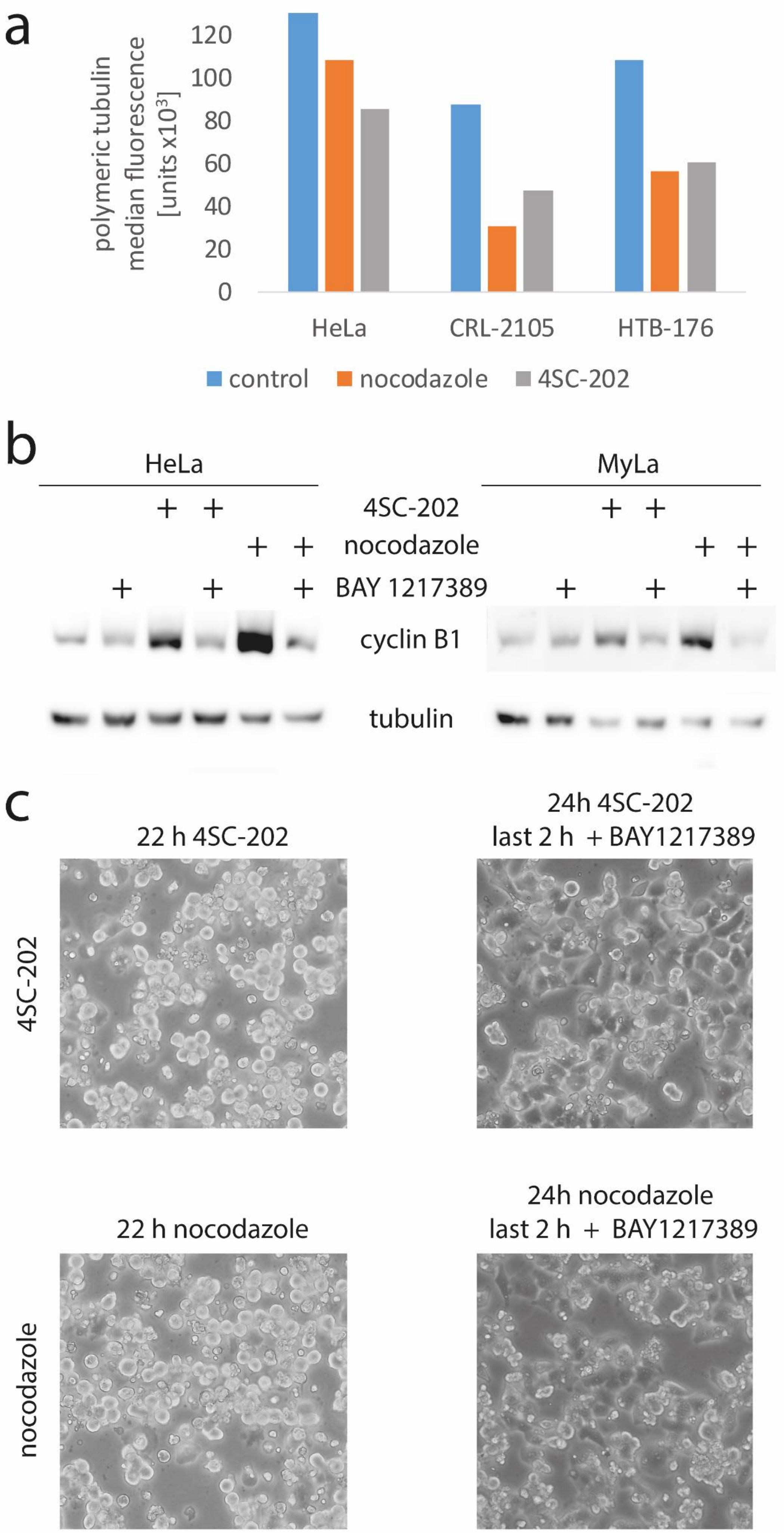

Supplementary Figure S12: 4SC-202 reduces the fraction of intracellular polymeric tubulin and activates the spindle assembly checkpoint. a) After 24 hours in the presence of 1 µM 4SC-202 or 100 nM nocodazole the indicated cells were subjected to tubulin staining under conditions where only polymerized tubulin is retained in the cells while tubulin monomers tend to get lost during the fixation procedure {Morrison, 2012 #1873}. **b and c)** After 22 hours in the presence of 1 µM 4SC-202 or 100 nM nocodazole an inhibitor specifically targeting the spindle checkpoint kinase MPS1 (BAY1217389; 10 nM) was added to the culture medium of the indicated cell lines. A BAY1217389-induced escape from mitotic arrest is evident in **b)** immunoblot analysis probing for Cyclin-B1 and by **c)** reattachment of detached mitotic HeLa cells.
